# Large‐scale forest restoration stabilizes carbon under climate change in Southwest United States

**DOI:** 10.1002/eap.1979

**Published:** 2019-08-16

**Authors:** Lisa A. McCauley, Marcos D. Robles, Travis Woolley, Robert M. Marshall, Alec Kretchun, David F. Gori

**Affiliations:** ^1^ Center for Science and Public Policy The Nature Conservancy Tucson Arizona 85719 USA; ^2^ Center for Science and Public Policy The Nature Conservancy Flagstaff Arizona 86001 USA; ^3^ Quantum Spatial Portland Oregon 97204 USA; ^4^ School of Natural Resources and the Environment University of Arizona Tucson Arizona 85721 USA

**Keywords:** climate change, forest carbon, forest restoration, LANDIS‐II, ponderosa pine southwest, wildfire

## Abstract

Higher tree density, more fuels, and a warmer, drier climate have caused an increase in the frequency, size, and severity of wildfires in western U.S. forests. There is an urgent need to restore forests across the western United States. To address this need, the U.S. Forest Service began the Four Forest Restoration Initiative (4FRI) to restore four national forests in Arizona. The objective of this study was to evaluate how restoration of ~400,000 ha under the 4FRI program and projected climate change would influence carbon dynamics and wildfire severity from 2010 to 2099. Specifically, we estimated forest carbon fluxes, carbon pools and wildfire severity under a moderate and fast 4FRI implementation schedule and compared those to status quo and no‐harvest scenarios using the LANDIS‐II simulation model and climate change projections. We found that the fast‐4FRI scenario showed early decreases in ecosystem carbon due to initial thinning/prescribed fire treatments, but total ecosystem carbon increased by 9–18% over no harvest by the end of the simulation. This increased carbon storage by 6.3–12.7 million metric tons, depending on the climate model, equating to removal of carbon emissions from 55,000 to 110,000 passenger vehicles per year until the end of the century. Nearly half of the additional carbon was stored in more stable soil pools. However, climate models with the largest predicted temperature increases showed declines by late century in ecosystem carbon despite restoration. Our study uses data from a real‐world, large‐scale restoration project and indicates that restoration is likely to stabilize carbon and the benefits are greater when the pace of restoration is faster.

## Introduction

In recent decades, the western United States has seen an increase in the frequency, size, and severity of wildfires (Dillon et al. 2011, Dennison et al. 2014). A century of fire suppression, along with a warmer climate, below‐average winter precipitation, and earlier spring snowmelt, has contributed to this increase (Abatzoglou and Williams 2016, Westerling 2016). This phenomenon is particularly pronounced in the southwestern United States, where the trend of warmer temperatures and extended droughts are projected to increase in the future. Climate‐induced changes are expected to lead to increased tree mortality due to drought stress, insect outbreaks, and larger and more severe wildfires (Williams et al. 2010, 2012, McDowell et al. 2015).

In addition to the economic costs, destruction of habitat, and air and water quality (Western Forestry Leadership Council 2010, Combrink et al. 2013), large wildfires can reduce carbon storage for decades after fire (Breshears and Allen 2002, Hurteau et al. 2008). There is an urgent need to accelerate the pace and scale of restoration in forests across the western United States to reduce vulnerability to catastrophic wildfire and improve resilience to climate change. As a response, the U.S. Congress authorized the Collaborative Forest Landscape Restoration Program (CFLRP) in the 2009 Omnibus Public Lands Management Act to accelerate restoration in high‐priority landscapes (U.S. Department of Agriculture, Forest Service 2015). The largest landscape to receive funding and begin implementation, referred to as the Four Forest Restoration Initiative (4FRI), is an effort to restore fire‐adapted ponderosa pine forests across four national forests in northern Arizona. Using a collaborative science‐based process, an environmental impact statement (EIS) for the first phase of 4FRI was approved in 2015, to restore significant portions of the Coconino and Kaibab National Forests (U.S. Department of Agriculture, Forest Service 2013). This project includes an initial restoration of thinning and prescribed fire followed by maintenance fire in subsequent years to maintain the conditions.

Restoration of forest structure (mechanical thinning) and processes (prescribed fire) is one of the few adaptation strategies available to forest managers. Because 4FRI's goal is to accelerate the pace of restoration across tens of thousands of hectares, 4FRI presents a rare opportunity to evaluate the extent to which restoration efforts aimed at restoring fire regimes can also be applied as mitigation strategies to stabilize carbon under climate change. Previous studies have projected that restoration under climate change will result in long‐term increases in forest carbon due to reductions in wildfire activity (Loudermilk et al. 2016, Hurteau 2017, Krofcheck et al. 2017, Liang et al. 2018). Mediated in part by low regeneration rates under warmer temperatures, other studies have projected that extreme climate change will result in forest type changes, biomass loss, and deforestation despite restoration treatments (Tarancón et al. 2014, Flatley and Fulé 2016, Loehman et al. 2018).

The objective of this study was to explore how the pace of restoration in ponderosa pine forests under a large‐scale restoration program would influence carbon dynamics and wildfire severity given projected changes in climate. Specifically, we estimated forest carbon fluxes and pools and wildfire severity under a moderate and rapid 4FRI implementation schedule and compared those to status quo restoration and no harvest using the LANDIS‐II simulation model. This study had several unique features that add value to previous efforts. It was one of the few that was conducted at a large‐scale and under an accelerated pace of restoration. Additionally, we incorporated U.S. Forest Service's (USFS) 4FRI implementation plan, with initial thinning prescriptions followed by maintenance fire into the LANDIS‐II model to match, as closely as possible, the actual restoration underway in the forests for the next several decades. Finally, we estimated the fate of carbon from harvested products and the resulting storage/emissions. In comparison to status quo restoration and no harvest, we hypothesized that ecosystem carbon would initially decline under accelerated thinning and prescribed fire but subsequently increase by reducing fuel loads available for wildfires under warmer and drier conditions caused by climate change.

## Methods

### Study area

**Figure 1:**
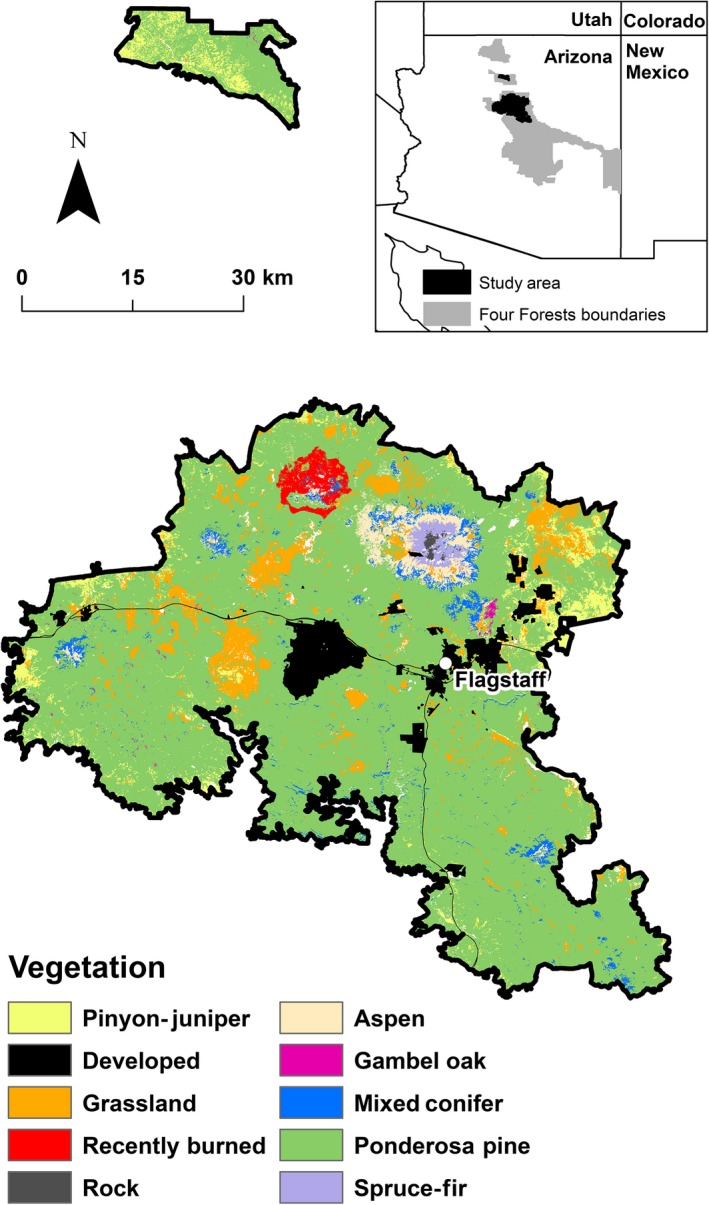
Location and vegetation of the 4FRI Phase I project area.

The study area was the initial phase of 4FRI, ~400,000 ha of the Coconino and Kaibab National Forests including the city of Flagstaff in northern Arizona, USA (Fig. 1). We included the entire footprint of 4FRI Phase 1 in our study but the actual 4FRI EIS did not include some areas within that footprint, as they already had separate, previously approved restoration plans that were similar in scope to 4FRI. We simulated restoration of the entire project area following the methods used by USFS for the 4FRI restoration.

The elevation of the study area ranges from 1,780 to 3,850 m with a mean elevation of 2,190 m. The average temperature is 10.2°C and average annual precipitation is ~460 mm. The 4FRI landscape is dominated by contiguous ponderosa pine tree cover intermixed with lesser amounts of dry and wet mixed conifer forests types and oak. Ponderosa pine forests in this area have shifted from naturally open conditions to high densities of small diameter trees in the last century, due to fire suppression, harvesting, and episodic regeneration events (Covington and Moore 1994).

### Scenarios

1. No Harvest/Control: In this scenario, we assumed no harvest or prescribed fire occurred in the project area for the entire length of the simulation. Wildfire was still simulated.
2. Status quo restoration: We estimated the current pace of restoration by averaging the area across which thinning and prescribed fire occurred in years 2010–2016. This included 1,200 ha of thinning and ˜3,600 ha of prescribed fire per year for a period of 20 yr followed by ˜3,600 ha of prescribed fire per year for the remaining 70 yr of the simulation (Table 1).
3. Moderate pace of restoration: This included 12,000 ha of thinning and 7,000 ha of prescribed fire per year, such that entire project area was treated in a period of 20 yr, followed by 7,000 ha of prescribed fire per year for the remaining 70 yr of the simulation (Table 1).
4. Fast pace of restoration: This was an accelerated pace of restoration that closely matched 4FRI's target restoration pace. It included restoring the same area as the moderate pace of restoration but in half the time; 24,000 ha of thinning and 14,000 ha of prescribed fire per year for the first 10 yr followed by 14,000 ha of prescribed fire per year for the remaining 80 yr of the simulation (Table 1).

## LANDIS‐II Model

The LANDIS‐II model is a spatially explicit landscape model that simulates forest succession and disturbances, and tracks tree growth using species‐age cohorts rather than individual trees (Scheller et al. 2007). Life history attributes are species‐specific model input parameters that inform how trees grow and compete. Species‐age cohort biomass is tracked in each active cell across the landscape. To simulate succession, we used LANDIS‐II v. 6.2 and the Net Ecosystem Carbon and Nitrogen (NECN) Succession extension (v. 4.2.4; formerly the Century Succession extension) that simulates monthly growth, mortality, and reproduction and also tracks carbon, nitrogen, and water cycling both above‐ and belowground (Scheller et al. 2011). For disturbances, we used the Biomass Harvest extension (v. 3.2.3) to simulate both thinning and prescribed fire and the Dynamic Fire and Dynamic Fuels extensions (v. 2.1) to simulate wildfire.

### GCM selection and climate inputs

We used daily precipitation and temperature data from randomly selected years from 1981 to 2010, from the Parameter‐elevation Relationships on Independent Slopes Model (PRISM; PRISM Climate Group, Oregon State University 2016) to run model spin‐up. Future climate projections were based on four global circulation models (GCMs) that were selected based on their correlation with historical climate data and their representation of climate extremes in the future. To do this, we calculated correlation coefficients of monthly PRISM vs. modeled historical (1981–2005) data from each GCM using four climate variables that previous studies have found to be important to ponderosa pine growth and recruitment (Feddema et al. 2013, Kolb et al. 2013, Petrie et al. 2016, 2017): winter precipitation, summer average temperature, spring average temperature, and summer relative humidity. Using those same four climate variables plus annual precipitation, we also calculated the difference between the averages from 1981 to 1999 and 2081 to 2099. We then choose four GCMs based on those that were highly correlated with the historical period and fell into each of these categories: below and above average increases in modeled temperature and below and above average changes in modeled precipitation. Chosen GCMs were INMCM4 (warm/dry), BCC‐CSM1‐1 (warmer/wetter), IPSL CM5A LR (hot/drier), MIROC ESM CHEM (hotter/wet) and were obtained from Multivariate Adaptive Constructed Analogs data sets (Appendix S1: Figs. S1, S2; Abatzoglou and Brown 2012). RCP 8.5 models were used in all cases. Monthly precipitation (mm), maximum temperature (°C), minimum temperature (°C), and the standard deviation of each of those variables were input into the NECN succession extension. Daily precipitation (mm), average temperature (°C), wind speed (km/h), wind direction (degrees), and relative humidity (%) were used to calculate a fire weather index, fine fuel moisture code, and build‐up index for use in the Dynamic Fire extension. Wind speed and wind direction were not expected to change with future climate change (Eichelberger et al. 2008), so historical daily wind direction and wind speed (Western Regional Climate Center 2018) were randomized and used for future climates. Future fire weather was input as different files for each decade so that fire weather would change into the future by decade.

**Table 1:** Time period, area treated, and fire return interval for each scenario

	Initial				Maintenance	
Scenario	Thinning period (yr)	Thinning area (ha)	Prescribed fire area (ha)	Area treated (ha)	Fire area (ha)	Fire return interval (yr)
Status quo	20	1,200	3,600	4,800 (1% of area)	3,600 (1% of area)	100
Moderate 4FRI	20	12,000	7,000	19,000 (5% of area)	7,000 (2% of area)	50
Fast 4FRI	10	24,000	14,000	38,000 (10% of area)	14,000 (4% of area)	25

*Notes*

The no‐harvest scenario is not included, as no treatments were made. Total project area is 386,100 ha.

### Initial communities

LANDIS‐II requires an initial communities input file that specifies the species and age cohorts for every cell on the landscape. We included 10 species in our model: ponderosa pine (*Pinus ponderosa*), aspen (*Populus tremuloides*), pinyon pine (*Pinus edulis*), juniper (we used four similar species in the genus *Juniperus* that all occur in the region), Gambel oak (*Quercus gambelii*), white fir (*Abies concolor*), subalpine fir (*Abies lasiocarpa*), Engelmann spruce (*Picea engelmannii*), blue spruce (*Picea pungens*), and Douglas fir (*Pseudotsuga menziesii*). We created a raster layer with 100‐m grid cells and used USFS inventory data from the 4FRI project (USFS, *unpublished data*) to assign an initial community to each cell. To do this, we developed diameter to age regressions for each of our species, based on USFS Forest Inventory and Analysis (FIA) data (U.S. Department of Agriculture, Forest Service 2017). We used the regressions to convert tree‐level 4FRI inventory data to ages and binned ages into 10‐yr bins. Unique species–age cohort combinations were created and assigned to every cell on the landscape. Our study area was divided into 14 ecoregions based on a combination of soil texture/depth information derived from U.S. Forest Service soil surveys (U.S. Department of Agriculture, Forest Service 1991, 1995) and four precipitation classes (PRISM Climate Group, Oregon State University 2016; Appendix S1: Fig. S3).

### NECN Succession

The NECN Succession extension simulates regeneration, growth, and mortality of trees, wood and litter decomposition, soil accumulation and decomposition, and available soil water (Scheller et al. 2011, 2012). NECN tracks all pools and fluxes of carbon and nitrogen as well as the biomass in each species/age cohort in all cells. NECN uses species and ecoregion parameters to define how cohort growth is affected by soil and climate (Data S1). Maximum biomass and maximum aboveground net primary productivity (ANPP) were defined for each species and each ecoregion. In addition to species‐specific parameters that outline many physiological characteristics (Data S1), NECN succession further organizes species into functional groups. This classification helps dictate tree growth responses to environmental conditions, with many parameters inherited from the Century model (Parton et al. 1993). Probability of establishment, which dictates the likelihood of successful regeneration, is the product of species‐specific traits and environmental and edaphic conditions. As cohorts grow, they compete for resources within each cell and can disperse across grid cells. Cohorts die when they reach species longevity values or are killed by disturbance (i.e., harvest or fire). Some species, functional group, and NECN parameters used in this study were taken from similar studies that used the same or similar species (Scheller et al. 2011, Loudermilk et al. 2013, Gustafson et al. 2015, Dymond et al. 2016, Hurteau et al. 2016).

### Wildfire simulation

We used the Dynamic Fuel extension to assign fuel types to the landscape based on dominant species/age structure in each 100‐m grid cell. Fuel types influence the spread and severity of wildfire on the landscape. Fuel types can change with every time step and are reassigned if species composition and ages change (via growth or disturbance) in a cell from the previous time step. We used 12 pre‐disturbance fuel types based on species and ages that were expected to burn similarly (Data S1). We also used five post‐disturbance fuel types that had increased crown base height, and thus lower fire severity, because we expected that treatment would alter the vertical structure of the trees on the landscape (Graham et al. 1999, Jain et al. 2007).

We used the Dynamic Fire extension to simulate wildfire on the landscape. We divided the landscape into three fire regions based on the probability of fire ignitions. Fire ignition probability was determined by reproducing an analysis from Dickson et al. (2006) using topographic roughness, presence of ponderosa pine, and precipitation as independent variables and historical fires >1 ha (Short 2017) as the dependent variable. Fire size distribution, seasonal foliar moisture content (FMC), and number of ignitions were input for each fire region (Data S1). Fire size distribution and number of ignitions for each fire region were obtained from Short (2017). FMC was calculated for each season (spring/summer/fall) using the latitude/longitude and average elevation of each ecoregion (Forestry Canada 1992), and day of the year for the beginning and end of each season based on local fire staff knowledge and fire database information (Short 2017). Fire weather index was also calculated at a daily time scale for the entirety of the simulation, using daily climate projections. Fire weather index was binned into severity classes (1–5), to provide specific weather information for future simulated fires. When running LANDIS‐II, fire occurrence is determined based on the number of ignitions for each fire region and the probability of fire initiation. We used the number of fires >1 ha from the fire database (Short 2017) as the number of ignitions in each fire region and the probability of fire initiation was determined by the dominant vegetation type. Fire spread in LANDIS‐II is determined by fire weather, topography, and fuel conditions (Sturtevant et al. 2009). Once a fire is initiated, the fire spreads until it reaches the predetermined fire size as selected from a lognormal distribution from user input μ (mean fire size) and σ^2^ (standard deviation of fire size). Future fire weather was randomly selected from a distribution of fire weather days from each decadal fire weather file. Likelihood of cohort mortality is based on the severity of the fire and the age class of the cohort; this is parameterized in the Dynamic Fire input text file (Data S1). For each age cohort that is killed in a fire, a user‐defined proportion remains on site as dead wood and a proportion is volatilized. These proportions for wood and litter vary by fire severity and are parameterized in the fire reduction table in the NECN Succession text file (Data S1).

### Management

We used the Biomass Harvest extension to simulate the 4FRI thinning prescriptions and prescribed fire prescriptions. Biomass Harvest requires the landscape to be divided into “management units,” within which targets are defined for the amount of area treated. We divided the landscape into six management units based on the prescriptions that were to be placed there. We developed 15 LANDIS‐II prescriptions based on the prescriptions used by USFS (*unpublished data*) in the 4FRI restoration project: grassland prescriptions that removed all trees; savanna prescriptions that removed most trees; nine prescriptions that removed ponderosa pine at various levels; and four prescribed fire prescriptions that included initial prescribed fire and maintenance prescribed fire for grasslands, savannas, and the remaining project area (Data S1). To realistically simulate current and anticipated management across the 4FRI landscape, we designed our management prescriptions to mimic the thinning practices used by managers, including age and diameter targets for individual species. Because LANDIS‐II is a cohort‐based model and cannot simulate individual tree removal, we then used expected pre‐ and post‐treatment condition 4FRI data from the USFS (*unpublished data*) to estimate the biomass removed from each cohort in each prescription in LANDIS‐II (Table 2). The Dynamic Fire extension is best suited for simulating wildfire effects, therefore we used the Biomass Harvest extension to simulate prescribed fires. As is designed for 4FRI, we implemented prescribed maintenance fires to continue after initial thinning and prescribed fire (10 or 20 yr, depending on scenario).

**Table 2:** Percentage of biomass removed with each prescription

Age class	Grassland^a^	Savanna^b^	IT10^c^	UEA10^c^	SI10^c^	IT25^c^	UEA25^c^	SI25^c^	IT40^c^	UEA40^c^	SI40^c^	Prescribed fire^d^
1	100	70	100	100	42	100	80	100	100	100	100	80
2	100	70	90	90	46	75	60	75	100	95	90	50
3	100	70	60	70	36	50	50	50	98	90	85	20
4	100	0	15	50	34	10	45	10	40	90	80	0
5	100	0	0	20	0	0	15	0	0	25	0	0
6	0	0	0	1	0	0	2	0	0	0	0	0

*Notes*

IT, intermediate thin; UEA, uneven age; SI, stand improvement. Numbers 10, 25, and 40 refer to the amount of openness (%) expected after thinning. Ages in each age class vary by species (see harvest input text files).

a All species except *Populus tremuloides*.

b Varies by species (see input text files).

c *Pinus ponderosa*,* Pseudotsuga menziesii*,* Abies concolor*. 100% removed in all age classes of *Abies lasiocarpa*,* Picea engelmannii*,* Picea pungens*.

d All species; initial and maintenance prescribed fire.

### Model calibration/validation

LANDIS‐II runs a model spin‐up for a length of time that is equal to the age of the oldest cohort, placing cohorts on the landscape in each time step corresponding to their current age. The oldest cohort in our initial communities layer was estimated to be 800 yr old, resulting in a model spin‐up time period of 800 yr. After model spin‐up, at year 0, we compared the biomass in each ecoregion from LANDIS‐II to the biomass at each ecoregion (Appendix S1: Fig. S4) from USFS data. LANDIS‐II biomass values were less variable than inventory data but the median LANDIS‐II biomass values in each ecoregion matched inventory data well, reliably falling within the interquartile range. As is common with LANDIS‐II, initial biomass values were slightly overestimated because spin‐up does not include disturbances (Scheller et al. 2018). The average biomass from the inventory data (11,396 g/m^2^) and the average biomass from LANDIS‐II calibrations at year 0 (14,931 g/m^2)^ were within expected bounds (Appendix S1: Fig. S4).

To calibrate the Dynamic Fire extension, we ran the simulations for 30 yr with historical climate input for growth and fire weather and compared the mean fire size, standard deviation of the fire size, fire return interval, and area burned per year for each fire region to fires from a fire database (Short 2017; Appendix S1: Table S1). We prioritized area burned per year over the other metrics in the calibration to ensure that it was similar to historical data. The average area burned across the project area in the historic database was 3,387 ha/yr and in the LANDIS‐II calibrations was 3,651 ha/yr (Appendix S1: Table S1). To calibrate the Biomass Harvest extension, we compared the biomass removed overall and from each prescription to USFS data (Appendix S1: Fig. S5).

### Simulations and analysis

We ran 10 replicate simulations for each scenario and each GCM from 2010 to 2099 using an annual time step for all extensions. We conducted sensitivity analyses by evaluating aboveground biomass output averaged for 2, 5, and 10 replicates and found little difference between averages of 5 and 10 replicates; thus, we ran 10 replicates of each scenario. For each scenario/GCM, we evaluated total ecosystem carbon (TEC), individual pools of carbon (i.e., live carbon, dead carbon, and soil carbon), net ecosystem production (NEP), wildfire emissions, wildfire severity and net ecosystem carbon balance (NECB) at each time step and averaged across replicates. NEP is gross primary productivity minus ecosystem respiration with positive values indicating the landscape is acting as a sink for carbon and negative values indicating a source of carbon to the atmosphere. NECB is NEP minus carbon lost from fire and prescribed fire, with positive values indicating the landscape is acting as a sink for carbon and negative values indicating a source of carbon to the atmosphere. NECB also often includes carbon lost from thinning but, in this study, a separate analysis outside of LANDIS‐II was used to determine the fate of carbon post‐thinning and thus, the carbon storage/emissions from thinning (see harvested‐products analysis below) was not included in our NECB estimates. TEC values reported are those output from LANDIS‐II and do not include carbon that remains sequestered in harvested material.

Fire severity class is created for each cell in each time step and ranges from 1 to 5 with 5 representing the most severe wildfire. We refer to fire severity classes as 1, low; 2, low–moderate; 3, moderate; 4, moderate–high; and 5, high. Fire severity was evaluated across time steps for each cell and compared across scenarios. Fire sizes were summed for each time step and averaged across replicates to compare among scenarios. We used ArcMap 10.3.1 (ESRI 2015) and R v 3.4.0 (R Development Core Team 2017) to conduct analyses and produce figures.

## Harvested‐products analysis

In addition to the models in LANDIS‐II, we developed and applied a simple flow analysis to determine the carbon storage and emissions during and following harvest through local product creation. Although not a comprehensive life cycle assessment (Oneil et al. 2017), we included emissions from harvesting equipment, emissions from transportation of wood (stem wood and biomass) to wood products facilities, emissions from biomass products that decompose in our analysis timeframe, and storage from wood products. To calculate carbon emissions from harvesting activities, we followed the methodology outlined by Markewitz (2006) combined with local operator and machine productivity rates for restoration thinning. To determine carbon emissions from transportation, we used local knowledge of individual truck load mass (stem wood and residual biomass separately), average distance traveled to product facilities (wood products and chipped wood separately), average fuel economy of transport vehicles, and U.S. Energy Information Administration's carbon dioxide emissions coefficients (U.S. Energy Information Administration 2016). To account for carbon storage and emissions from resulting forest products and waste associated with this production, we made several key assumptions. (1) The approximate amounts of wood products were as follows: pallets, 55%; single family homes (post 1980), 20%; manufacturing, 5%; railroad ties, 15%; and furniture, 5% (Stephen Horner, *personal communication*) and each product had a carbon half‐life as indicated in Skog and Nicholson (1998), (2) Chipped wood/biomass used for soil amendments was an emission for the length of analysis assuming decomposition rates for that material of ~10%/yr (Harmon et al. 1996). (3) Any residual biomass material created from processing wood products was an emission for the length of our analysis. We used local industry knowledge of proportions of wood vs. soil amendment products and the proportions of waste created when making wood products. Additional information can be found in Appendix S2.

## Results

**Figure 2:**
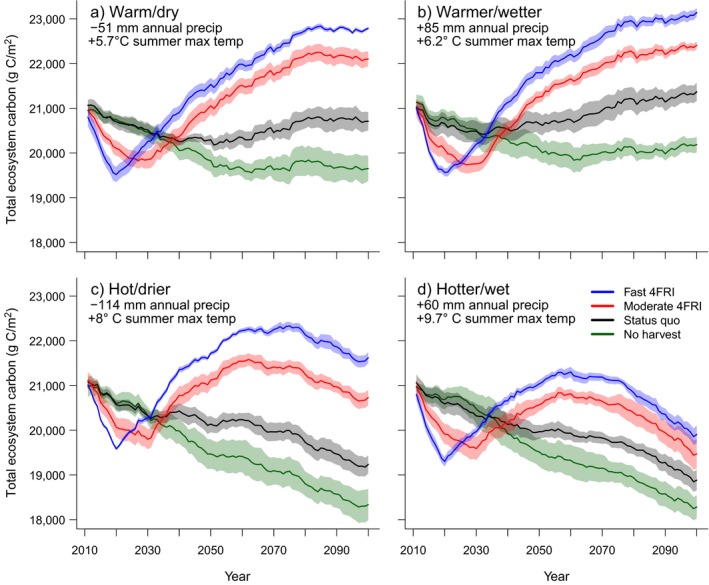
Total ecosystem carbon (TEC) as output from LANDIS‐II for the four climate models throughout the simulation model period. These values do not include estimates from carbon that remains sequestered in harvested products. Climate models are (a) INMCM (warm/dry); (b) BCC‐CSM1‐1 (warmer/wetter); (c) IPSL CM5A LR (hot/drier); (d) MIROC ESM CHEM (hotter/wet). Values of change in precipitation (precip) and temperature (temp) are changes from the observation‐based 1981–1999 average to the modeled 2081–2099 average. Shaded areas represent 95% confidence intervals.

Despite initial decreases in carbon in the first two decades due to accelerated harvest and prescribed fire, the moderate‐ and fast‐4FRI scenarios resulted in greater carbon storage by the end of the century than the status quo and no‐harvest scenarios and that pattern remained consistent among the climate models (Fig. 2). Depending on the climate model, the overall increases in TEC for the fast‐4FRI scenario were 9–18% higher than the no‐harvest scenario, equating to an increase of 6.3 million–12.7 million metric tons of carbon across the 4FRI project area. Climate models that predicted the largest temperature increases by late century showed greater decreases in TEC in all restoration scenarios by the end of the century (Fig. 2c, d).

**Figure 3:**
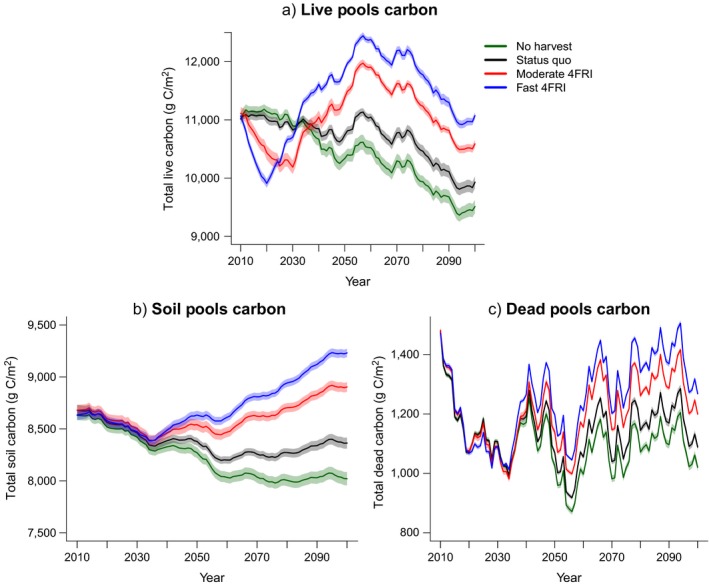
Pools of carbon that constitute total ecosystem carbon (TEC) throughout the simulation model period for the hot/drier climate model (IPSL CM5A LR). (a) Total live carbon includes aboveground (wood and leaves) and belowground (coarse and fine roots) live carbon; (b) total soil carbon includes all soil organic matter pools; (c) total dead carbon includes dead wood, dead leaves, and dead roots. Shaded areas represent 95% confidence intervals.

**Figure 4:**
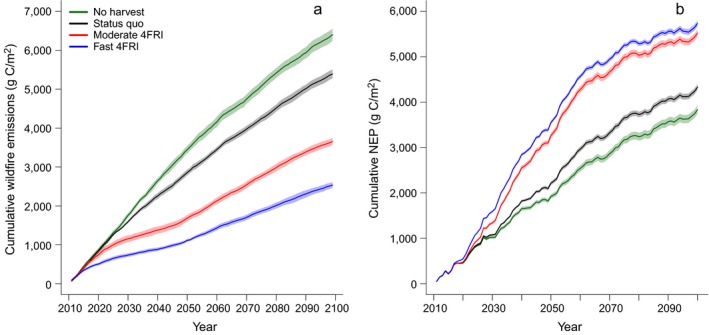
(a) Cumulative wildfire emissions from each scenario throughout the simulation model period. (b) Cumulative net ecosystem production (NEP) from each scenario throughout the simulation model period. Both are results from the hot/drier climate model (IPSL CM5A LR). Shaded areas represent 95% confidence intervals.

**Figure 5:**
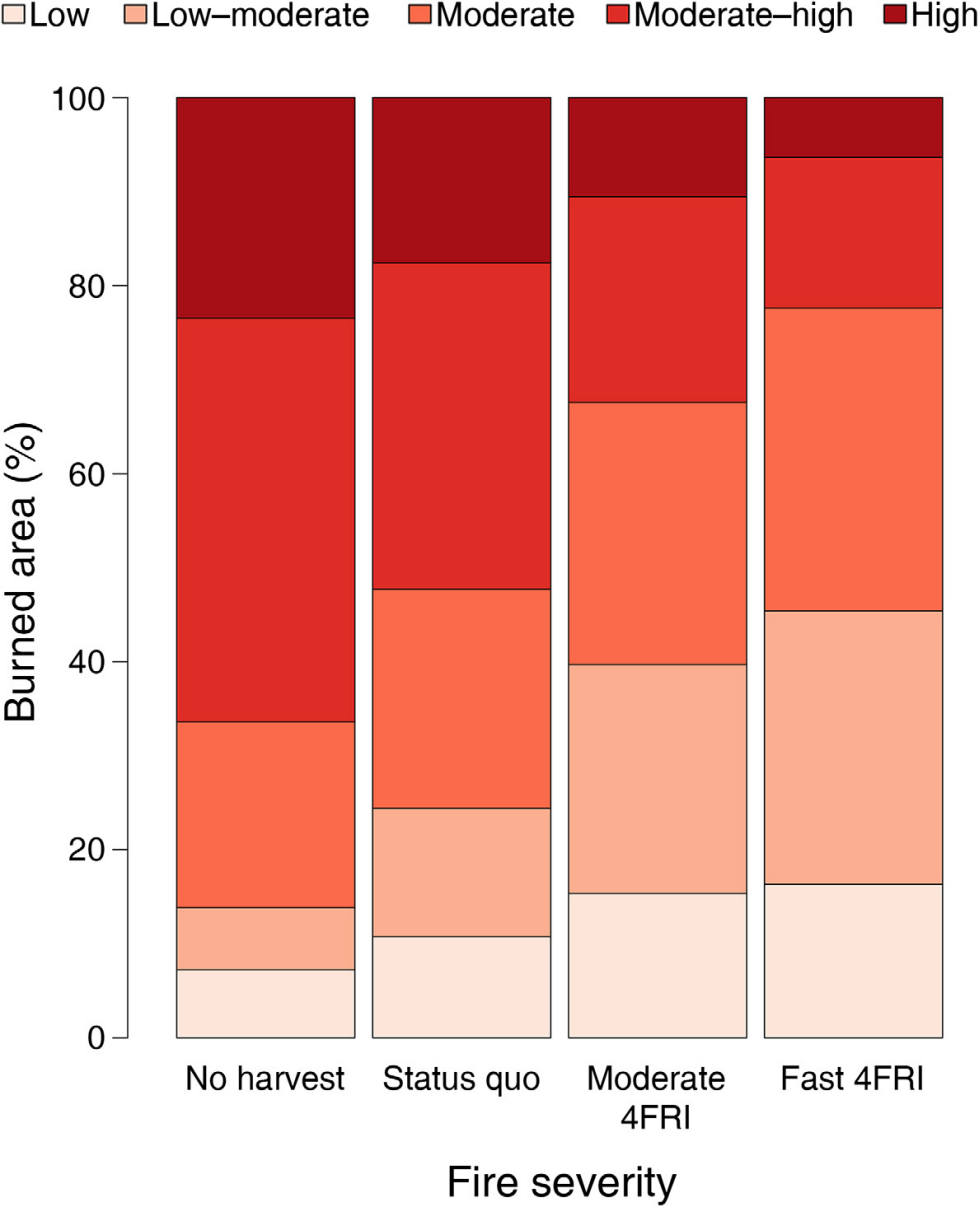
Percentage of burned area in each fire severity class in each scenario, averaged across all replicates and all years, for the hot/drier climate model (IPSL CM5A LR).

Given that the relative changes in carbon pools and fluxes in the four climate models were similar, we present the remaining results only for the hot/drier climate model here because it resulted in intermediate carbon estimates. Results from additional models can be found in the supplementary materials. Under the hot/drier climate model, live carbon pools accounted for 52%, soil carbon pools accounted for about 40%, and dead carbon pools accounted for about 8% of the overall TEC differences between the fast‐4FRI and the no‐harvest scenario at the end of the simulation (Table 3, Fig. 3; Appendix S1: Fig. S6). By 2099, the no‐harvest scenario had 1.5 times more wildfire emissions and 33% less production than the fast‐4FRI scenario (Fig. 4; Appendix S1: Figs. S7, S8). In terms of carbon fluxes, ~70% of the TEC increase in the fast‐4FRI relative to the no‐harvest scenario was explained by decreased wildfire emissions while greater production explained ~30%. In the fast‐4FRI scenario, ~22% of the area burned was burned at a high severity (moderate–high or high) compared to ~66% in the no‐harvest scenario (Fig. 5; Appendix S1: Fig. S9). There were no significant differences in annual area burned among the scenarios (Appendix S1: Fig. S10).

**Table 3:** Metric tons of carbon in each pool and the difference between the 4FRI fast and no‐harvest scenarios at year 90 of the simulation for the hot/drier climate model (IPSL CM5A LR)

Pool	No harvest	Status Quo	Moderate 4FRI	Fast 4FRI	Difference between 4FRI fast and no harvest
Live C	36,725,368	38,337,336	40,875,693	42,746,550	6,021,182
Soil C	30,965,845	32,290,974	34,375,518	35,642,006	4,676,161
Dead C	3,940,765	4,204,368	4,632,461	4,897,718	956,953
Harvest	–	9,445	82,149	69,313	69,313
Total carbon storage	70,793,157	74,274,574	80,125,315	83,548,453	12,755,296

*Notes*

The harvest pool represents the carbon that remained sequestered in wood products at the end of the simulation period.

**Figure 6:**
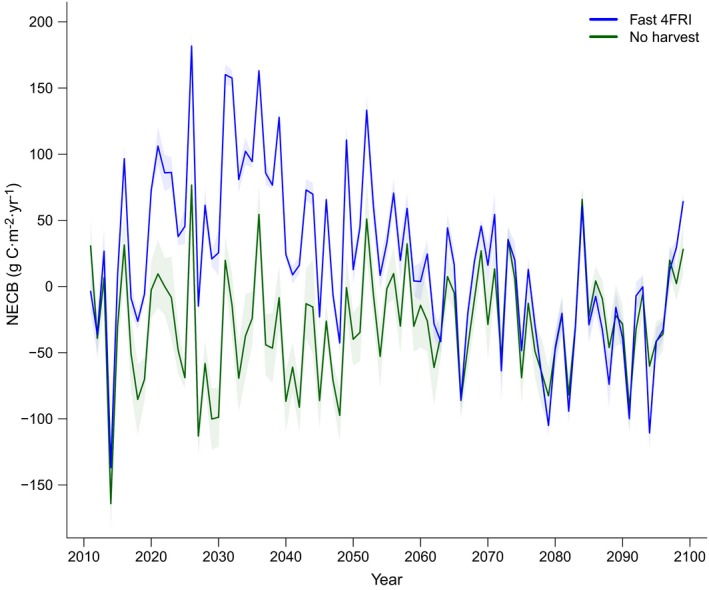
Net ecosystem carbon balance (NECB) for the 4FRI‐fast and no‐harvest scenarios throughout the simulation model period for the hot/drier climate model (IPSL CM5A LR). Shaded areas represent 95% confidence intervals.

NECB exhibited high interannual variability but was consistently higher in the fast‐4FRI scenario than in the no‐harvest scenario for approximately 50 yr after initial treatment, indicating that an accelerated harvest schedule can increase carbon sink strength (Fig. 6; Appendix S1: Fig. S11). Annual NECB values between the two scenarios converged in the last 40 yr when perhaps warmer temperatures and drier conditions in the hot/drier climate model (Appendix S1: Fig. S1) neutralized the effects of restoration.

The harvested‐products analysis showed that ~5% of the harvested carbon remained sequestered in wood products at the end of our study, adding an additional ~53 g C/m^2^ or 207,000 metric tons of carbon in the fast 4FRI scenario. In contrast, ~137,000 metric tons of carbon was emitted by the harvesting equipment and vehicles transporting the material off site. Thus, there was only a net gain of 70,000 metric tons of carbon from the harvested material, which represents only a fraction (~0.5%) of the carbon gains modeled in LANDIS‐II from the fast‐4FRI scenario (Table 3). Because these gains were so small, we did not include them in the overall total carbon storage for the landscape.

## Discussion

Our results demonstrate that large‐scale forest restoration can increase the potential for carbon storage and stability and those benefits could increase as the pace of restoration accelerates. The fast‐4FRI scenario could increase total carbon storage by ~12.7 million metric tons over the no‐harvest scenario in the hot/drier climate model, equating to the removal of the annual emissions from 110,000 passenger vehicles or the electricity consumed in over 90,000 homes per year until the end of the century. Increases in ecosystem carbon were greater when the 4FRI program was completed in 10 yr vs. 20 yr.

Potential gains in TEC in the fast‐4FRI scenario compared to the no‐harvest scenario were the result of greater productivity, explaining ~30% of the increases, and a reduction in wildfire emissions, explaining ~70% of the increases. Production increases were apparent in the 4FRI scenarios subsequent to initial thinning and prescribed fire (10 or 20 yr, depending on the scenario) and growth continued to remain higher in the 4FRI scenarios for ~50 yr. Additionally, smaller proportions of the landscape burned at high severity as the pace of restoration increased, resulting in lower tree mortality (Fig. 5). In the case of ponderosa pine, for example, the low and low‐moderate fire severity categories that occurred more frequently in the 4FRI scenarios killed only trees <10 yr old, whereas the moderate–high and high severity categories, that occurred more frequently in the no‐harvest scenario, killed all trees <80 and <340 yr old (nearly all the trees in most sites), respectively.

Climate change effects on forest carbon were most apparent in the last half of the century and these effects were more pronounced in the climate models that predicted hotter temperatures (Fig. 2). In the hot/drier and hotter/wet climate models, ecosystem carbon decreased in the last half of the century likely because higher temperatures and/or less precipitation led to lower rates of growth and establishment (Appendix S1: Fig. S12) that could not keep pace with increased mortality from wildfire (Appendix S1: Fig. S13), maintenance prescribed fire, and tree senescence. This effect was greatest in the hotter/wet climate model that predicted a 9.7°C increase in summer temperature by the end of the century, suggesting that a ~10°C increase in summer temperature could represent a threshold where the effects from restoration are diminished or eliminated. This is demonstrated in Fig. 4b, where the slopes of the lines representing cumulative NEP for 4FRI scenarios and the no‐harvest scenario become similar in the last 30 yr of the century, and in Fig. 6, where annual NECB values become similar and overall negative during the same time frame.

While no other studies have incorporated the level of detail to represent an actual landscape‐scale forest restoration project as we did, other studies have found similar results indicating that forest restoration can increase carbon storage and decrease wildfire severity (Loudermilk et al. 2014, 2016, Hurteau et al. 2016, Hurteau 2017, Krofcheck et al. 2017, 2018, Liang et al. 2018). Because 4FRI prescriptions were developed to achieve heterogeneity in size‐class distributions across a large landscape, the prescriptions in this study were more varied than the basic fuels reduction treatments in previous studies (Table 2). Previous studies have removed as little as 30% of live biomass focused on the smaller size classes (Hurteau et al. 2016, Krofcheck et al. 2017, e.g., Hurteau 2017), but in some cases removed up to 95% of post‐settlement trees (Flatley and Fulé 2016) or examined different thinning intensities and objectives (Loudermilk et al. 2014).

Still other studies found that, despite restoration efforts, extreme climate change conditions could result in significant biomass losses and deforestation (Tarancón et al. 2014, Flatley and Fulé 2016, Loehman et al. 2018). We also saw decreases in TEC and aboveground biomass in the no‐harvest and status quo scenarios implying that without restoration there is a strong likelihood that forest biomass will decline with climate change due to high severity fires and low productivity. Additionally, our study found lower recruitment across all scenarios with extreme increases in temperature (Appendix S1: Fig. S12), implying that, despite restoration efforts, biomass loss is still possible. We found similar vegetation type changes as Loehman et al. (2018) in a similar time frame but Flatley and Fulé (2016) found that vegetation type changes are possible further into the future.

However, when comparing our study to other studies, even those done with LANDIS‐II in the same region, we found widely varying assumptions and inputs and it is unknown what effect these had on the results. Without sensitivity analyses to evaluate the effect of different inputs, it is difficult to compare across studies leaving systematic review or meta‐analyses difficult (see James et al. 2018). Future research would benefit from more transparency in model assumptions and limitations and in reporting a common set of metrics for all studies.

We conducted a separate analysis evaluating the fate of carbon from the harvested products in this region. LANDIS‐II assumes all harvested material is removed from the ecosystem and we believe that assumption is valid in this region with the current wood product types. Our results suggest that there is little net gain in carbon from harvested material because the amount of carbon sequestered in wood products at the end of the study timeframe was roughly equivalent to the emissions from the equipment and transport vehicles used during the harvest. Most of the wood products in this region were pallets (55%) with a carbon half‐life of only 6 yr but if the harvested biomass were converted to longer‐lasting wood products, we would expect that this number would substantially increase (Finkral and Evans 2008). We did not include harvest in our estimates of NECB, but in accounting for the emissions from harvest, we would expect the NECB values for the fast 4FRI landscape would decrease by ~45 g C·m^−2^·yr^−1^ for the first 20 yr, including immediate emissions (material burned at processing) and decomposition over time (chipped and residual biomass). This indicates that the 4FRI landscapes were likely a greater source of carbon to the atmosphere during the first 20–30 yr than shown in the LANDIS‐II results (Fig. 6).

### Model limitations

We found that ~40% of the increases in carbon storage occurred in soil carbon, a stable long‐term carbon pool that is less vulnerable to losses from forest disturbances (Scheller et al. 2011). In LANDIS‐II, thinning, prescribed fire, and wildfire all add biomass to the dead pools of carbon on the landscape. Dead‐pool carbon quickly decomposes to become soil organic carbon and remains in soil carbon pools long term. However, there are limitations to the harvesting process used in LANDIS‐II because equal amounts of aboveground and belowground live carbon are removed from the system. Instead of transferring the belowground live carbon (roots) to the dead pools and eventually soil pools, the model removes the belowground pools from the ecosystem in the same way as aboveground live pools. This leads to fewer inputs into the dead pools and thus, fewer inputs into soil organic carbon, and a conservative estimate of the amount of carbon being stored in the soil. Additionally, black carbon is not accounted for in LANDIS‐II so it is not known how this would vary by scenario.

While the pace of restoration influenced wildfire severity in this study, neither restoration nor climate change significantly affected area burned per year (Appendix S1: Fig. S10). We believe this is an artefact of how the LANDIS‐II Dynamic Fire extension fits wildfire sizes to a lognormal distribution that cannot be altered with changing climatic conditions. This distribution shortens the right‐side tail of the fire size distribution in such a way that large wildfires that have already occurred and larger fires that are predicted to occur (Kitzberger et al. 2017, Keyser and Westerling 2019) are not simulated. However, despite the limitations of LANDIS‐II to model it, warming‐induced increases in wildfire size will likely occur (Westerling 2016) and result in higher carbon emissions. Restoration treatments, including prescribed fire, will remain important for reducing fire severity, but may not consistently reduce wildfire size (Cochrane et al. 2012, Price et al. 2015).

The purpose of 4FRI is to reestablish forest structure to improve forest health and the resiliency of forests to climate change, fire and other disturbances (U.S. Department of Agriculture, Forest Service 2013). Other studies have found that large‐scale forest restoration projects are likely to have co‐benefits that include maintaining biodiversity (Hurteau et al. 2014), improving water supply (Robles et al. 2014), improving insect resistance (Fettig et al. 2013, Kolb et al. 2016), and increasing soil function (Sánchez Meador et al. 2017). This study illustrates that large‐scale forest restorations are also likely to have potential co‐benefits of increased carbon storage, but climate change could diminish the effects of restoration if higher predicted temperatures are realized. We conclude that the 4FRI project will increase potential carbon stores in ponderosa pine forests by reducing tree mortality from severe wildfires and sustaining productivity under adverse climatic conditions. Our results also suggest that carbon increases can result from initial thinning followed by maintenance fires. Prescribed fire is less expensive and easier to implement than thinning and can provide benefits not achieved with thinning alone (North et al. 2012, Hood et al. 2015) and both are required to achieve the desired results. 4FRI is only one of 16 western U.S. landscapes in which the CFLR program has funded accelerated restoration of dry forests across 1.1 million ha (U.S. Department of Agriculture, Forest Service 2018). However, the scale of the fire risk is much larger: of the estimated 25 million ha of frequent‐fire conifer forests on federal lands in the western United States (Ager et al. 2013), fire regimes have been significantly altered and restoration is needed on more than 11 million ha (Menakis et al. 2004). All 11 million ha are likely not suitable for mechanical thinning. However, even if only one‐half are suitable and accelerated restoration in those landscapes has similar effects on the carbon cycle as 4FRI, substantial increases in carbon sequestration and stabilization at the sub‐continental scale could be realized. While accelerated forest restoration on its own is unlikely to be a sufficient long‐term response to climate change, carbon stabilization from accelerated restoration would buy time to retain forest cover, understand climate effects and develop management strategies that reduce the loss of forest cover from climate‐induced impacts. Short of enhancing the current CFLR program or developing new programs that address the urgent need to accelerate restoration, this study and others project potentially dramatic changes in forest carbon pools and forest cover in the Western United States.

## Data Availability

Data are available from Zenodo: http://doi.org/10.5281/zenodo.3265319
