# Supplementary material for: Large‐scale forest restoration stabilizes carbon under climate change in Southwest United States
Source: Ecol Appl. 2019 Aug 16;29(8):e01979. doi: 10.1002/eap.1979 (PMC6916600; doi:10.1002/eap.1979)
Supplement: Supplementary file 1 [file EAP-29-na-s001.pdf]

AppendixS1

Lisa A. McCauley, Marcos D. Robles, Travis Woolley, Robert M. Marshall, Alec Kretchun, and David F. Gori.
Large-scale forest restoration stabilizes carbon under climate change in Southwest United States
Ecological Applications

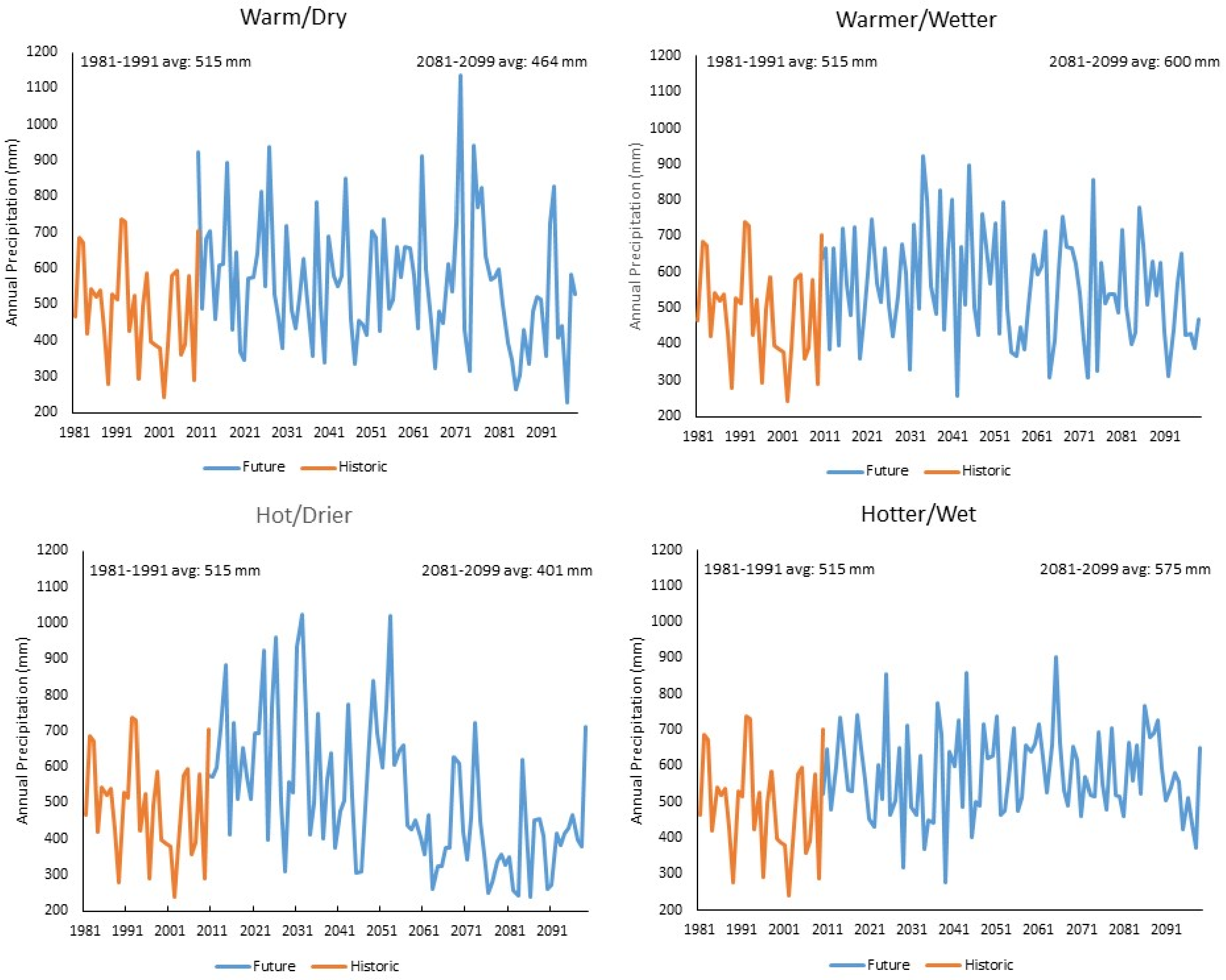

Figure S1. Annual precipitation values (mm) from 1981-2099 for each of the four climate models. Historic values are from observation-based PRISM climate data and the future values are from each of the climate models. GCM models are INMCM4 (warm/dry) , BCC-CSM1-1 (warmer/wetter), IPSL CM5A LR (hot/drier), MIROC ESM CHEM (hotter/wet)

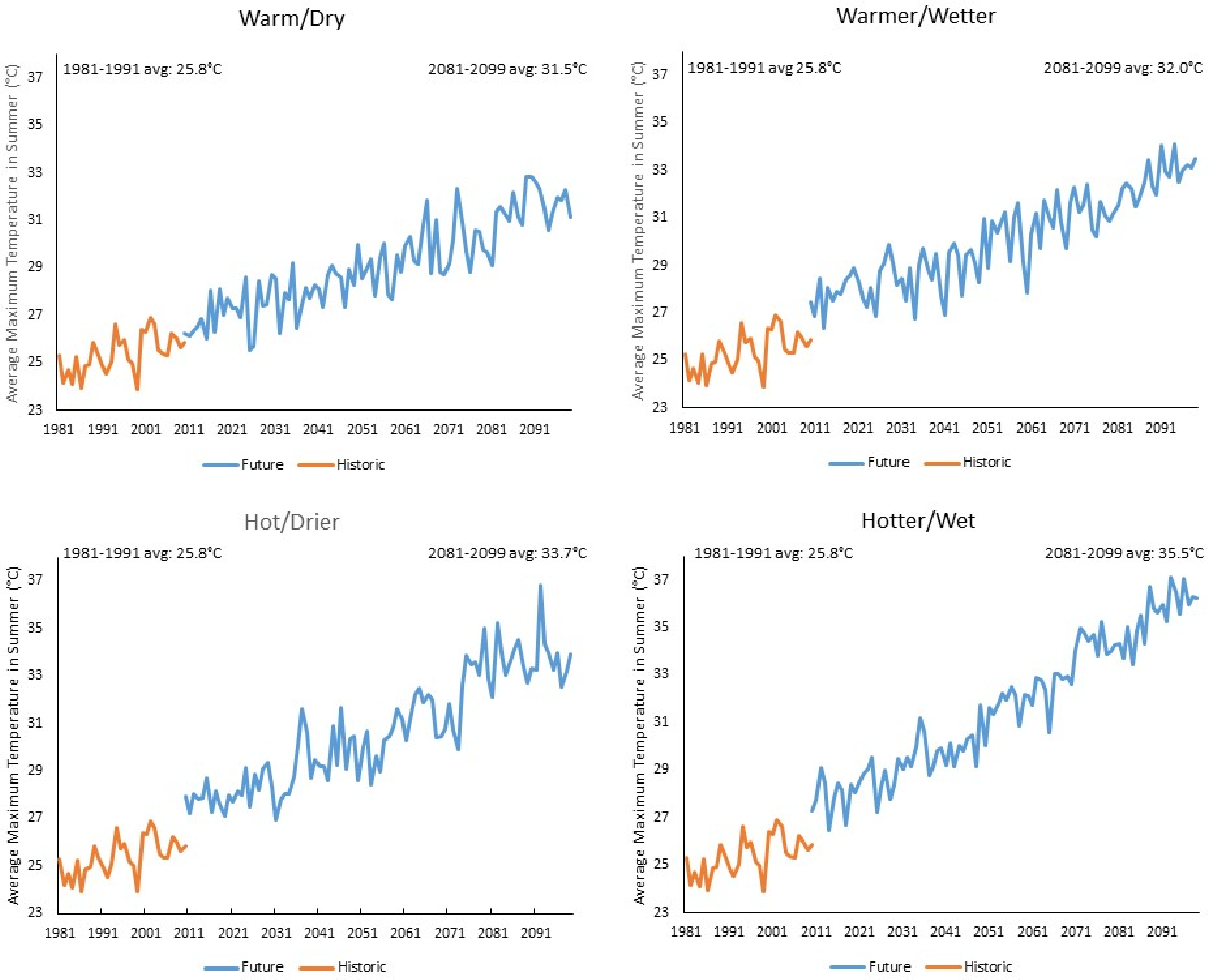

Figure S2. Average summer (June-September) temperature values from 1981-2099 for each of the four climate models. Historic values are from observation-based PRISM climate data and the future values are from each of the climate models.

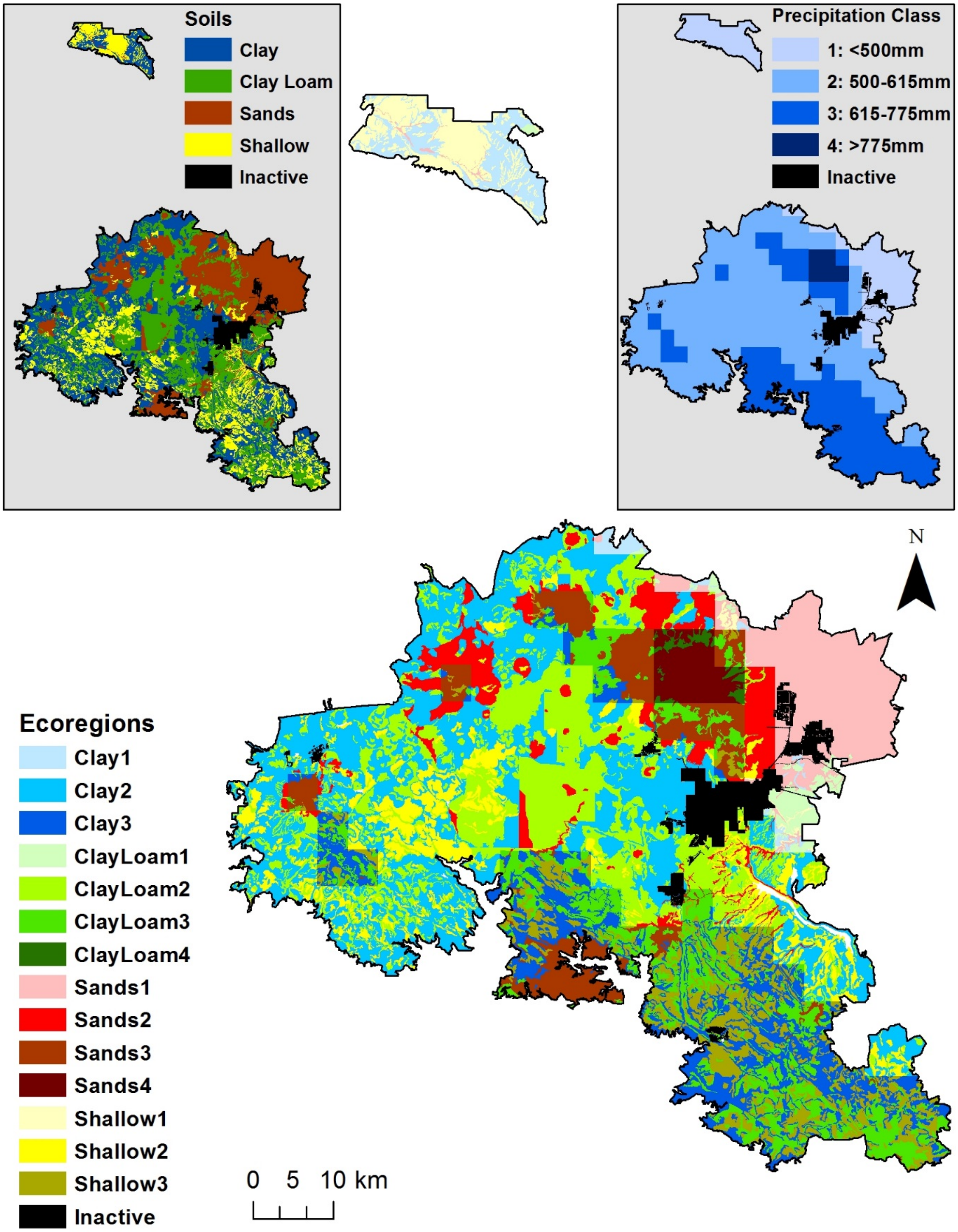

Figure S3. Ecoregion boundaries used in the Landis-II model. Ecoregions are a combination of soil texture classes and precipitation classes (inset maps).

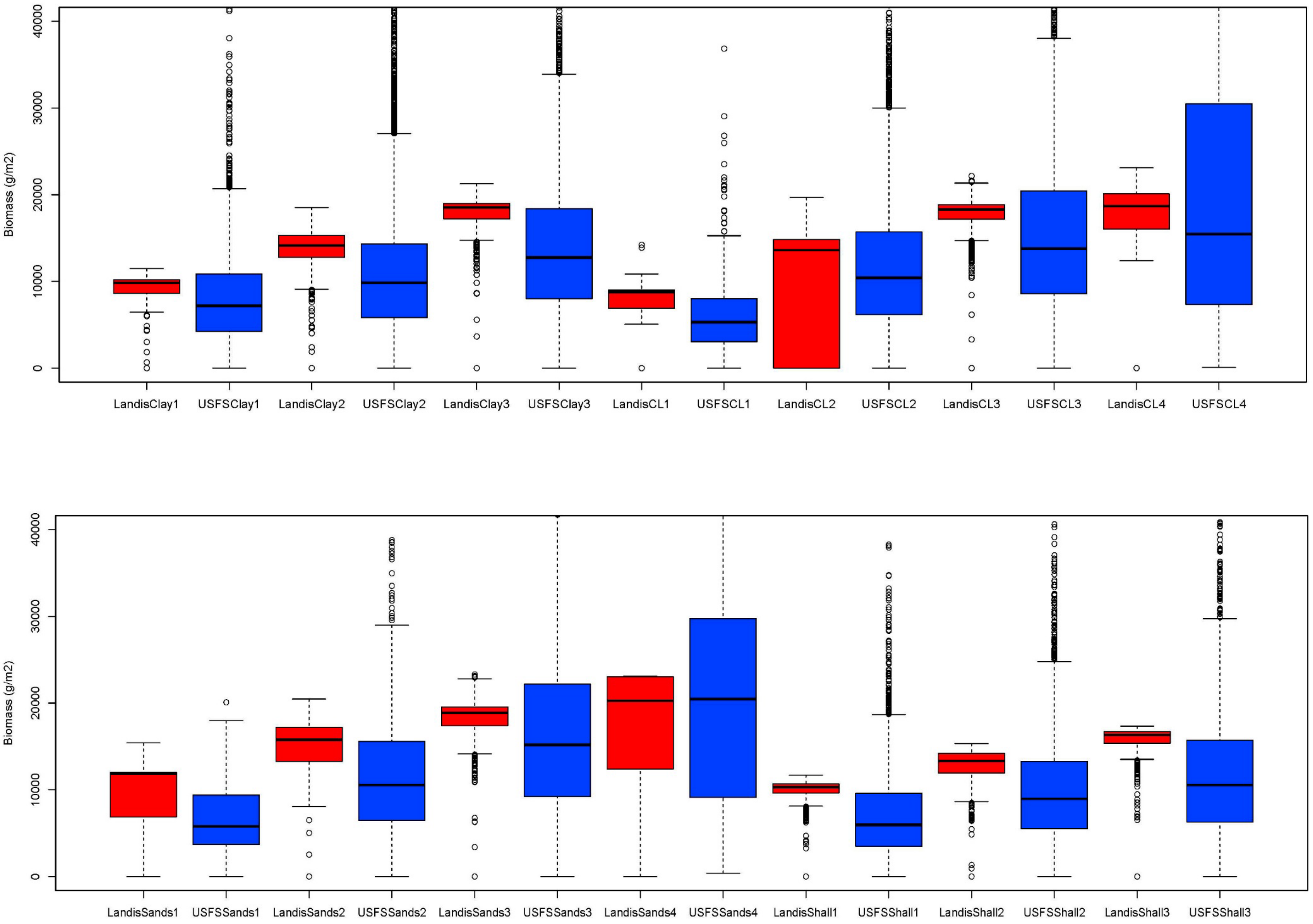

Figure S4. Boxplots of Landis-II-estimated (red) and USFS-estimated (blue) biomass (g/m2) in each ecoregion. Boxes represent the inter-quartile range, bold lines represent median, whiskers represent minimum and maximum values, and dots are considered outliers.

Fire Database Data					
Fire region	Mean fire size (ha)	St. Dev fire size (ha)	# fires / year	Fire return interval	Total area burned (ha)/year
Low	377.4	3,892.6	1.38	154	185
Moderate	425.6	3,968.8	6.46	80	1,872
High	375.5	7,250.2	8.21	65	1,330
Total Project Area	392.8	5,037.2	5.4	90	**3,387**

LANDIS-II Calibration					
Fire region	Mean fire size (ha)	St. Dev fire size (ha)	# fires / year	Fire return interval	Total area burned (ha)/year
Low	181.2	143.22	2.03	129	369
Moderate	252.1	222.30	6.00	93	1,513
High	214.9	208.74	8.23	120	1,770
Total Project Area	216.1	191.4	5.4	110	**3,651**

Table S1. Comparison of fire metrics from the fire database (Short 2017) and the LANDIS-II calibration. Area burned per year (bold) was prioritized over other fire metrics as the LANDIS-II metric that should match the data from the fire database best.

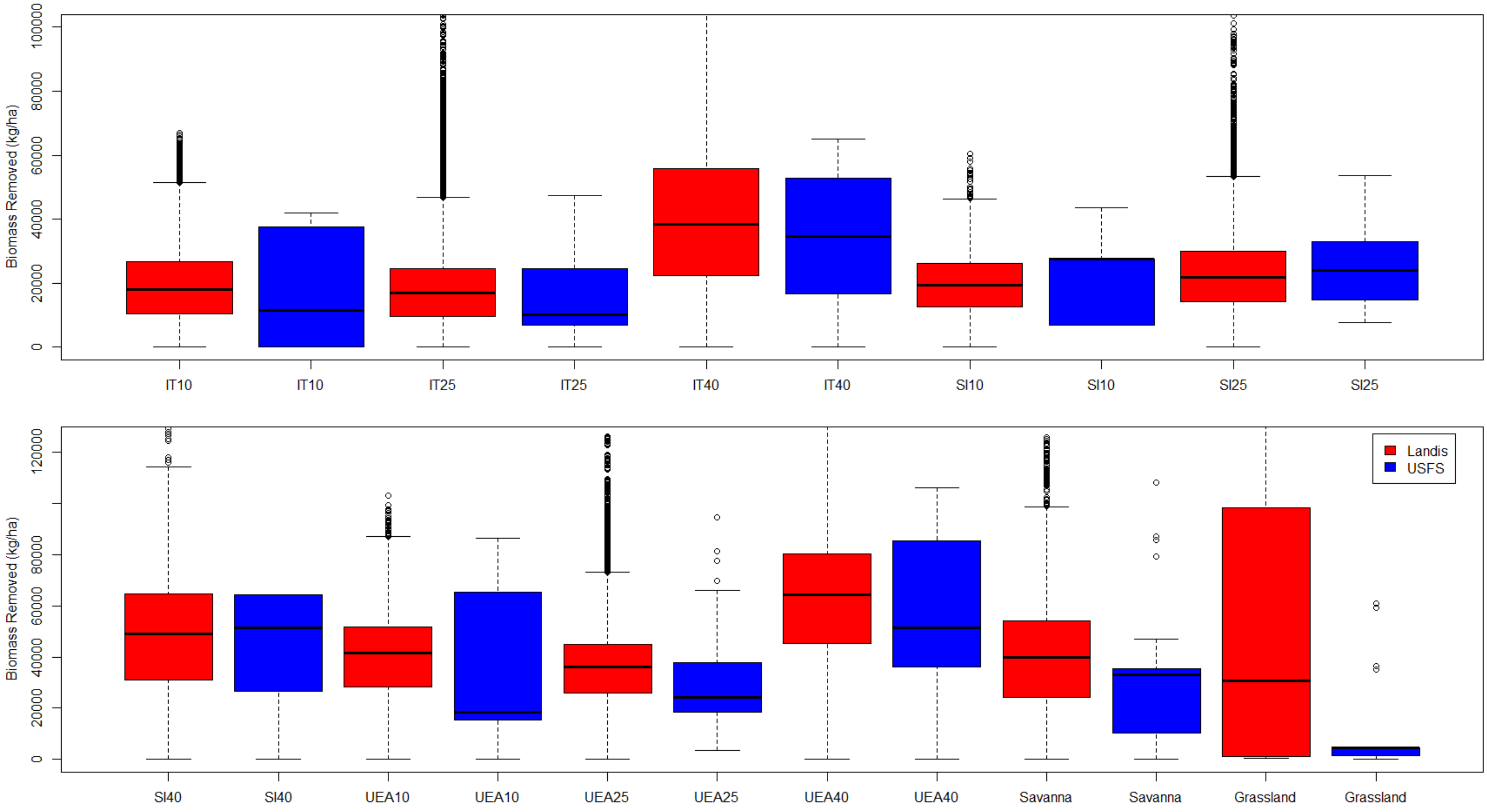

Figure S5. Boxplots of Landis-II-estimated (red) and USFS-estimated (blue) biomass removed (kg/ha) in each prescription. Boxes represent the inter-quartile range, bold lines represent median, whiskers represent minimum and maximum values, and dots are considered outliers.

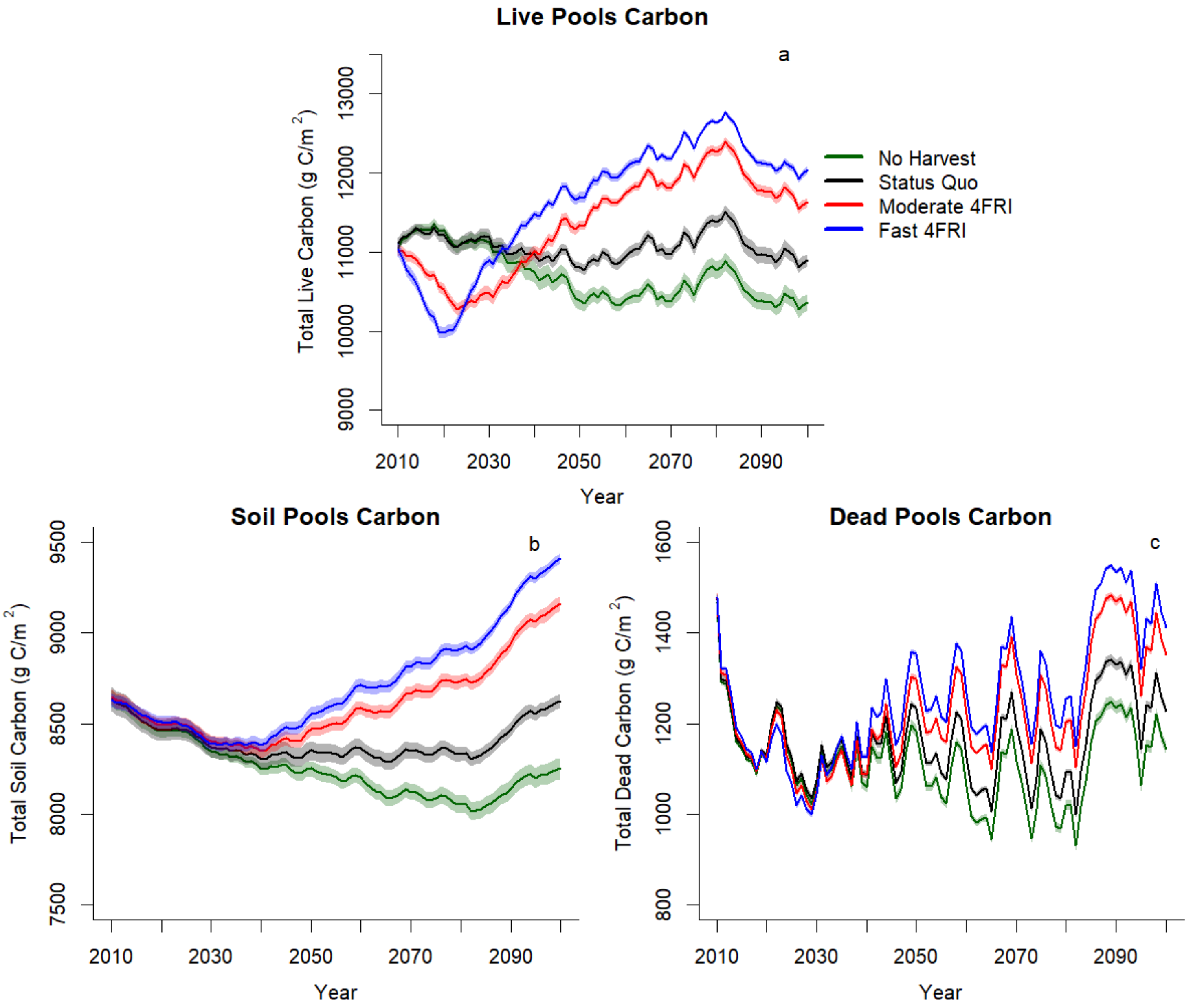

Figure S6a. Pools of carbon that constitute TEC throughout the simulation model period for the warm/dry climate model (INMCM4). a – Total live carbon (g C/m^2) includes aboveground (wood and leaves) and belowground (coarse and fine roots) live carbon; b – Total soil carbon (g C/m^2) includes all soil organic matter pools; c – Total dead carbon (g C/m^2) includes dead wood, dead leaves, and dead wood. Shaded areas represent 95% confidence intervals.

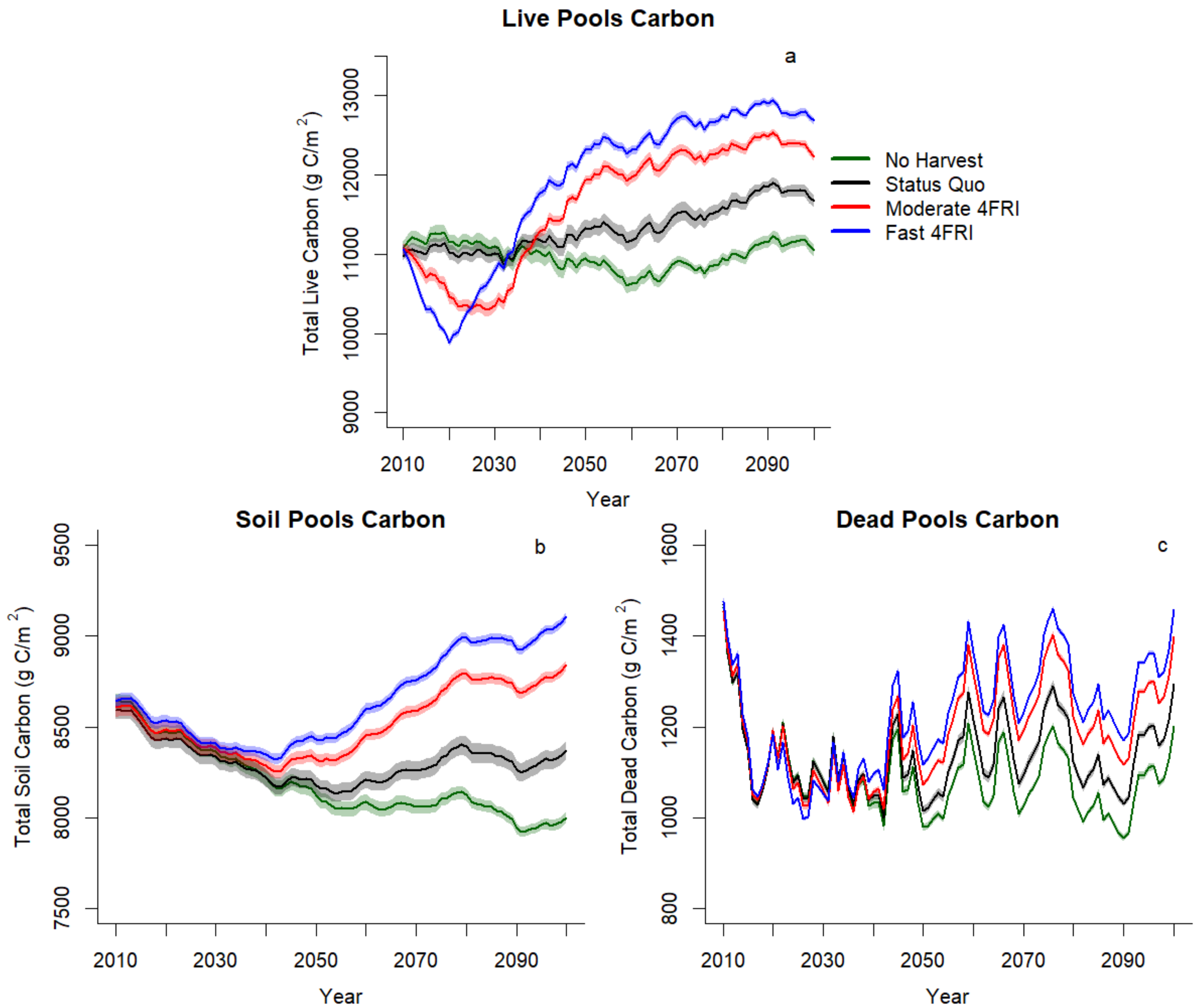

Figure S6b. Pools of carbon that constitute TEC throughout the simulation model period for the warmer/wetter climate model (BCC-CSM1-1). a – Total live carbon (g C/m^2) includes aboveground (wood and leaves) and belowground (coarse and fine roots) live carbon; b – Total soil carbon (g C/m^2) includes all soil organic matter pools; c – Total dead carbon (g C/m^2) includes dead wood, dead leaves, and dead wood. Shaded areas represent 95% confidence intervals.

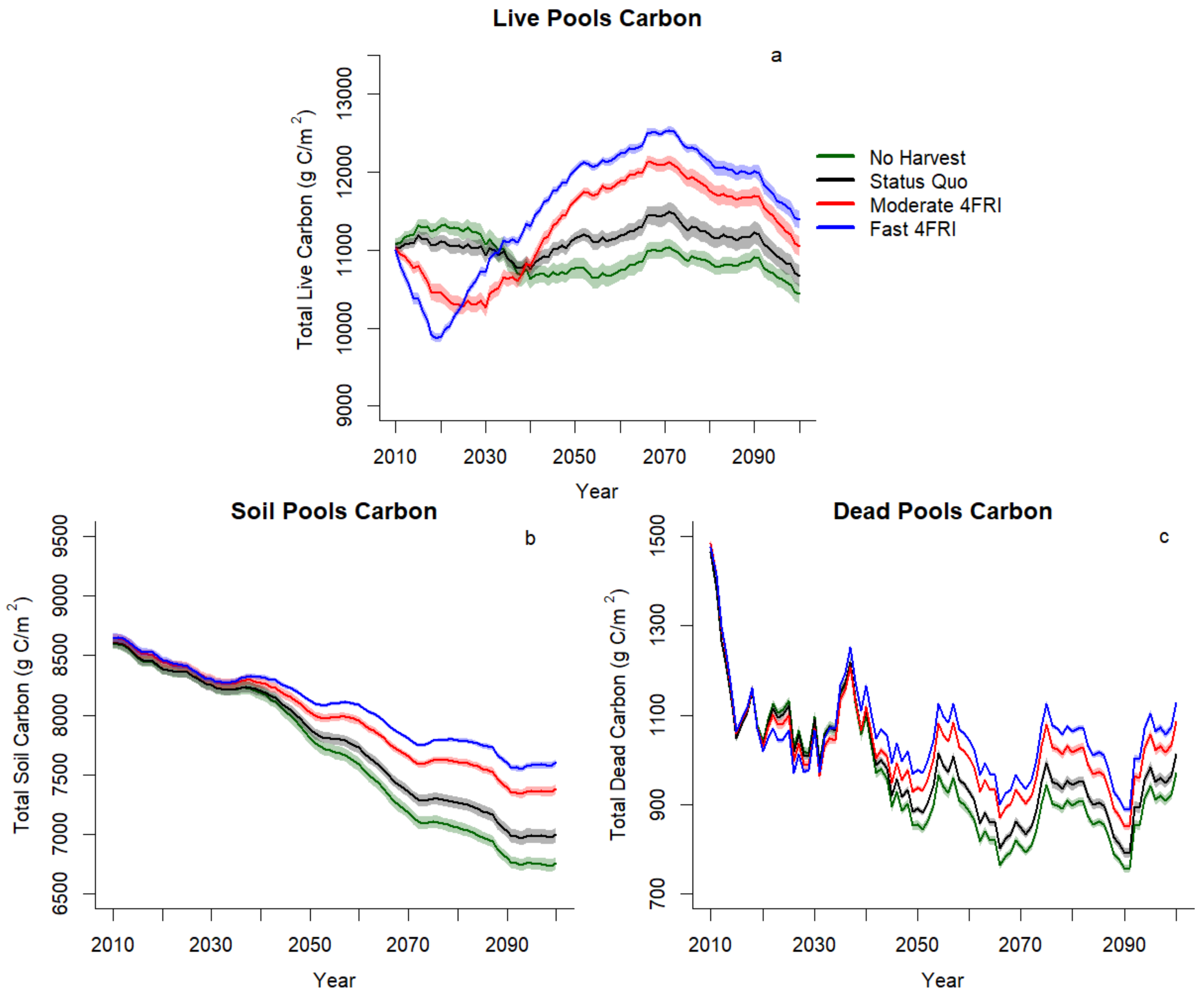

Figure S6c. Pools of carbon that constitute TEC throughout the simulation model period for the hotter/wetter climate model (MIROC ESM CHEM). a – Total live carbon (g C/m^2) includes aboveground (wood and leaves) and belowground (coarse and fine roots) live carbon; b – Total soil carbon (g C/m^2) includes all soil organic matter pools; c – Total dead carbon (g C/m^2) includes dead wood, dead leaves, and dead wood. Shaded areas represent 95% confidence intervals.

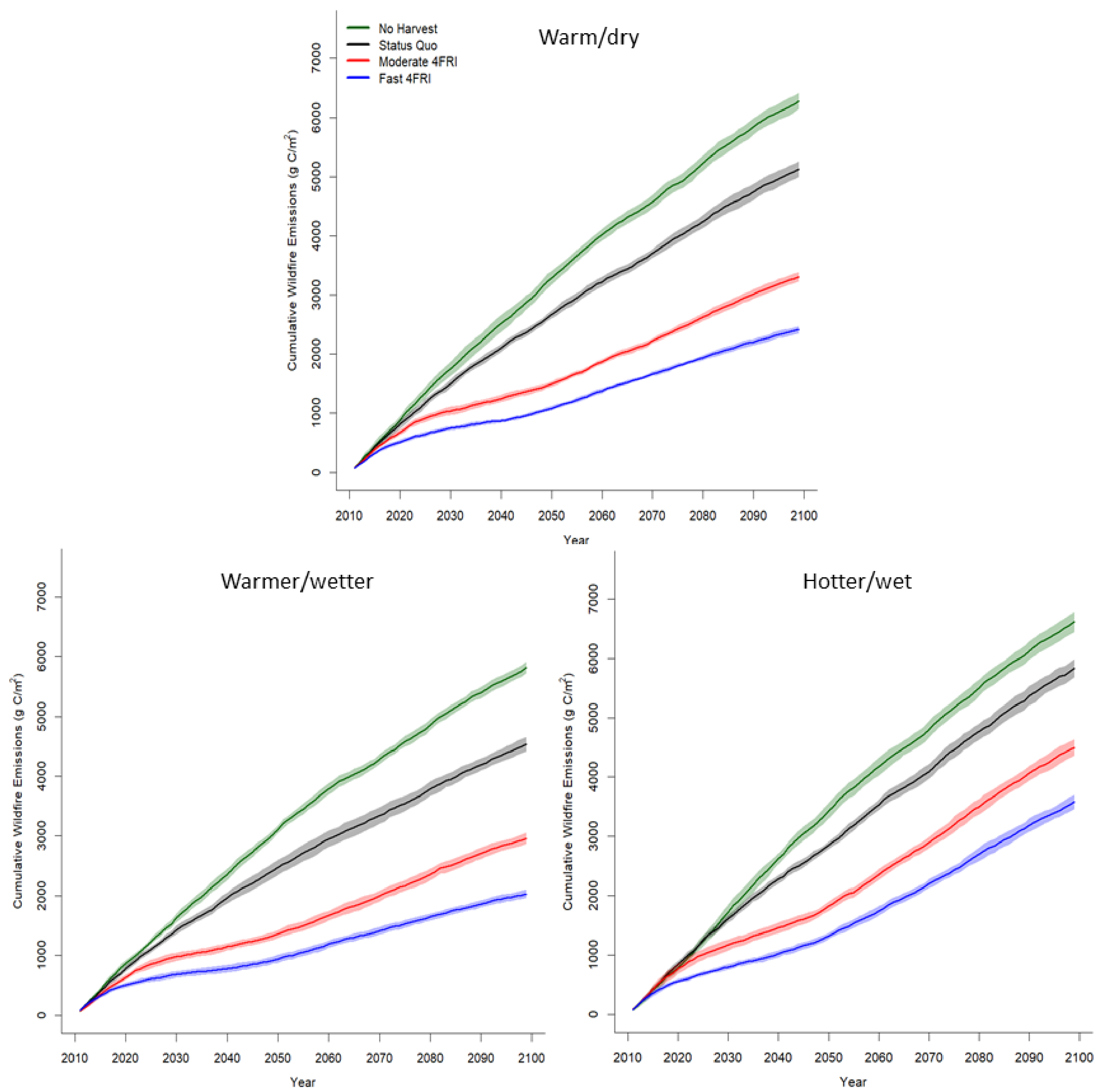

Figure S7. Cumulative wildfire emissions (g C /m^2) from each scenario throughout the simulation model period for each climate model. The hot/drier climate model can be found in the main paper. Shaded areas represent 95% confidence intervals.

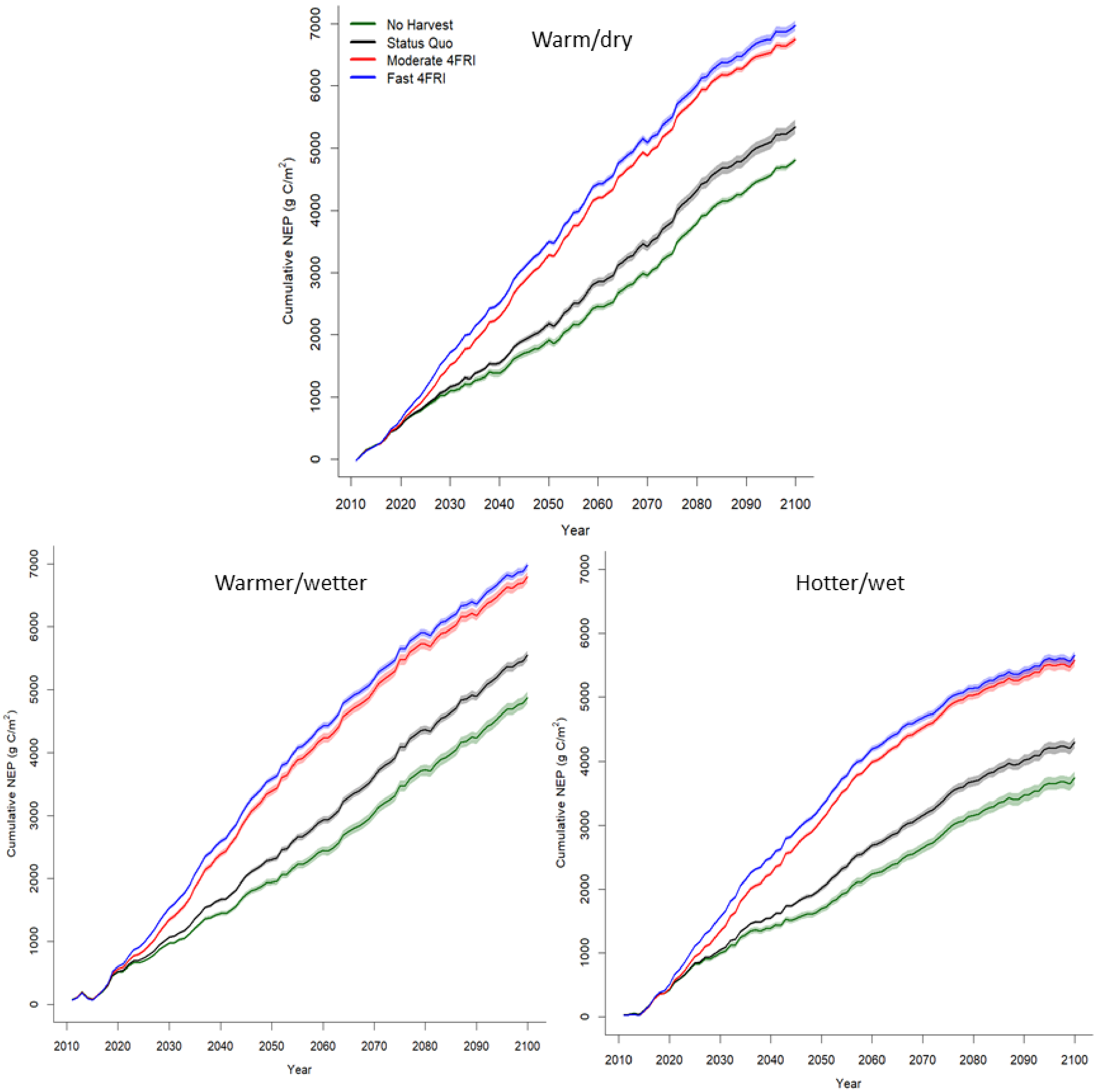

Figure S8. Cumulative NEP (g C/m^2) from each scenario throughout the simulation model period for each climate model. The hot/drier climate model can be found in the main paper. Shaded areas represent 95% confidence intervals.

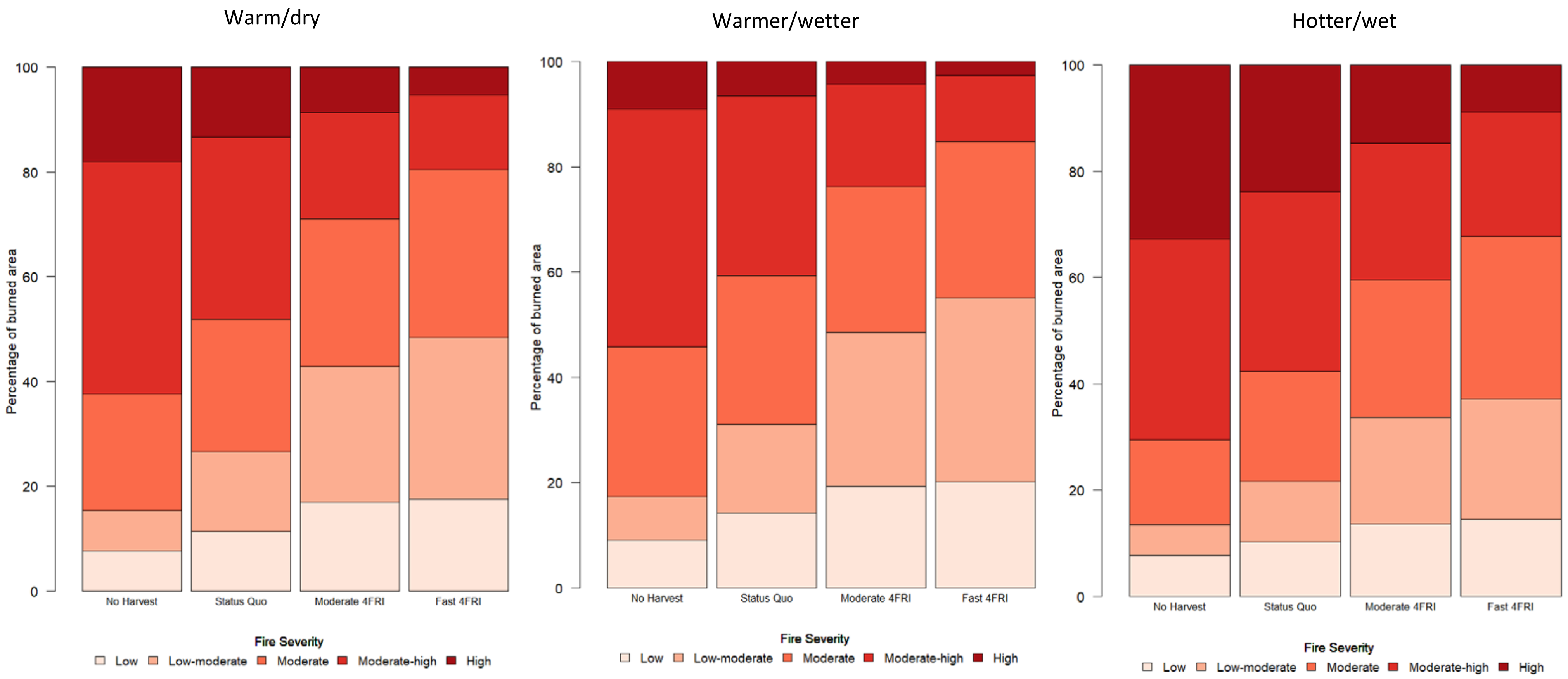

Figure S9. Percentage of burned area in each fire severity class in each scenario, averaged across all replicates and all years, for each climate model. The hot/drier climate model can be found in the main paper.

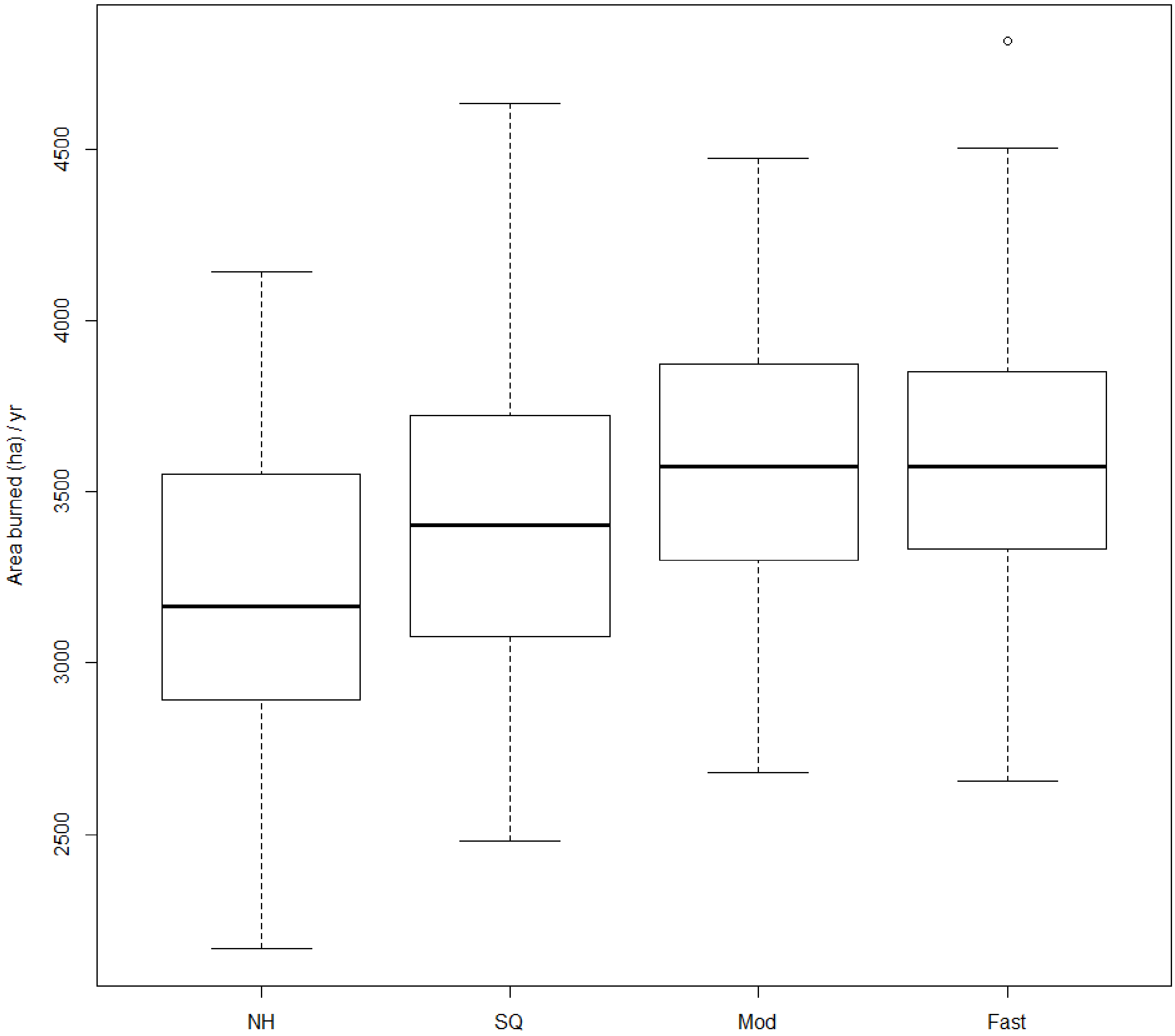

Figure S10. Area burned (ha) per year in each scenario for the hot/drier climate model. NH – No harvest; SQ – Status Quo; Mod – 4FRI Moderate; Fast – 4FRI Fast

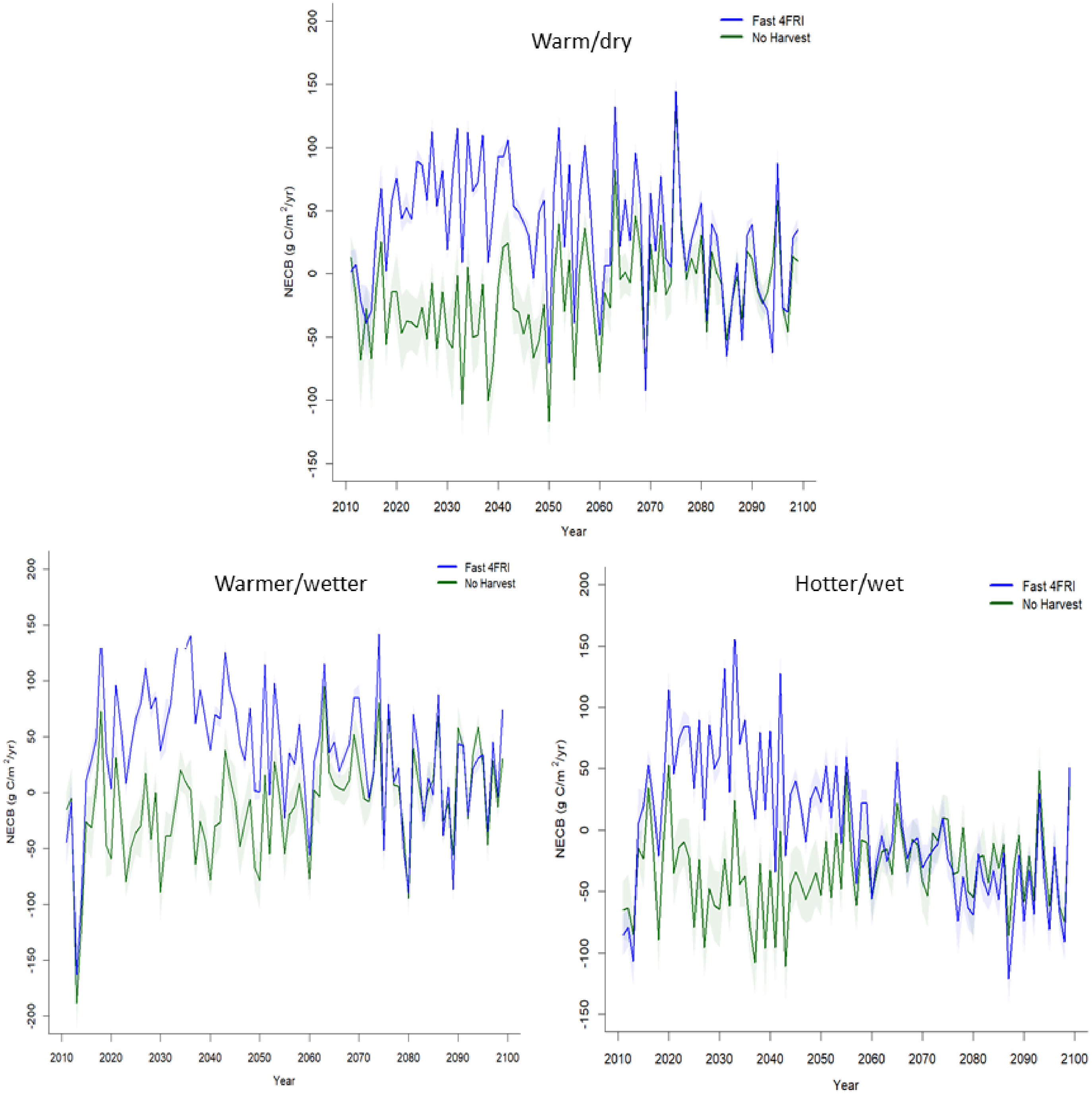

Figure S11. NECB (g C/m^2/yr) for the 4FRI-fast and no-harvest scenarios throughout the simulation model period for each climate model. The hot/drier climate model can be found in the main paper. Shaded areas represent 95% confidence intervals.

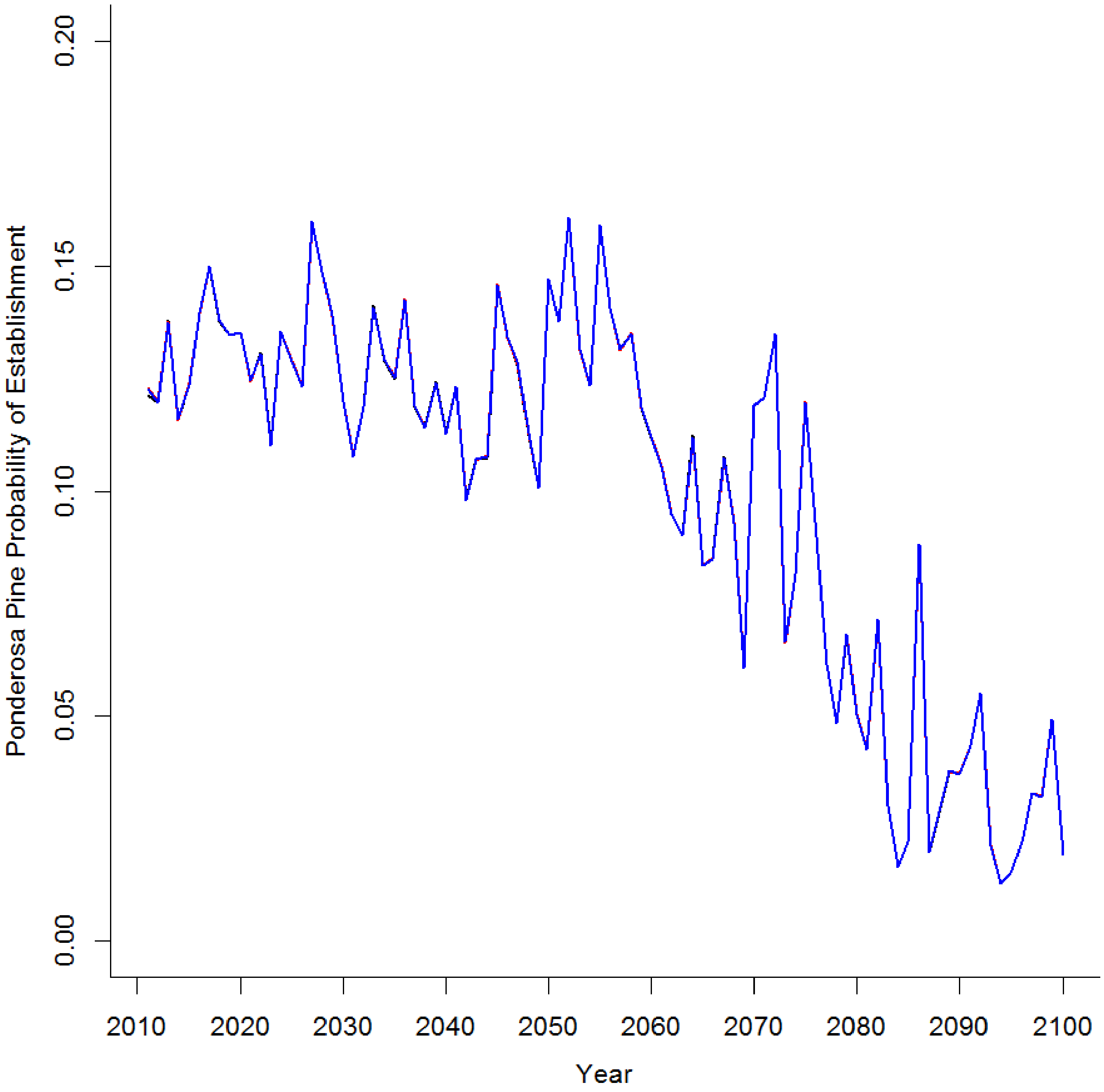

Figure S12. The probability of establishment of ponderosa pine averaged across all replicates through time, , for the hot/drier climate model (IPSL CM5A LR). All 4 scenarios are drawn as separate lines but with very little differences between lines, they are not all visible.

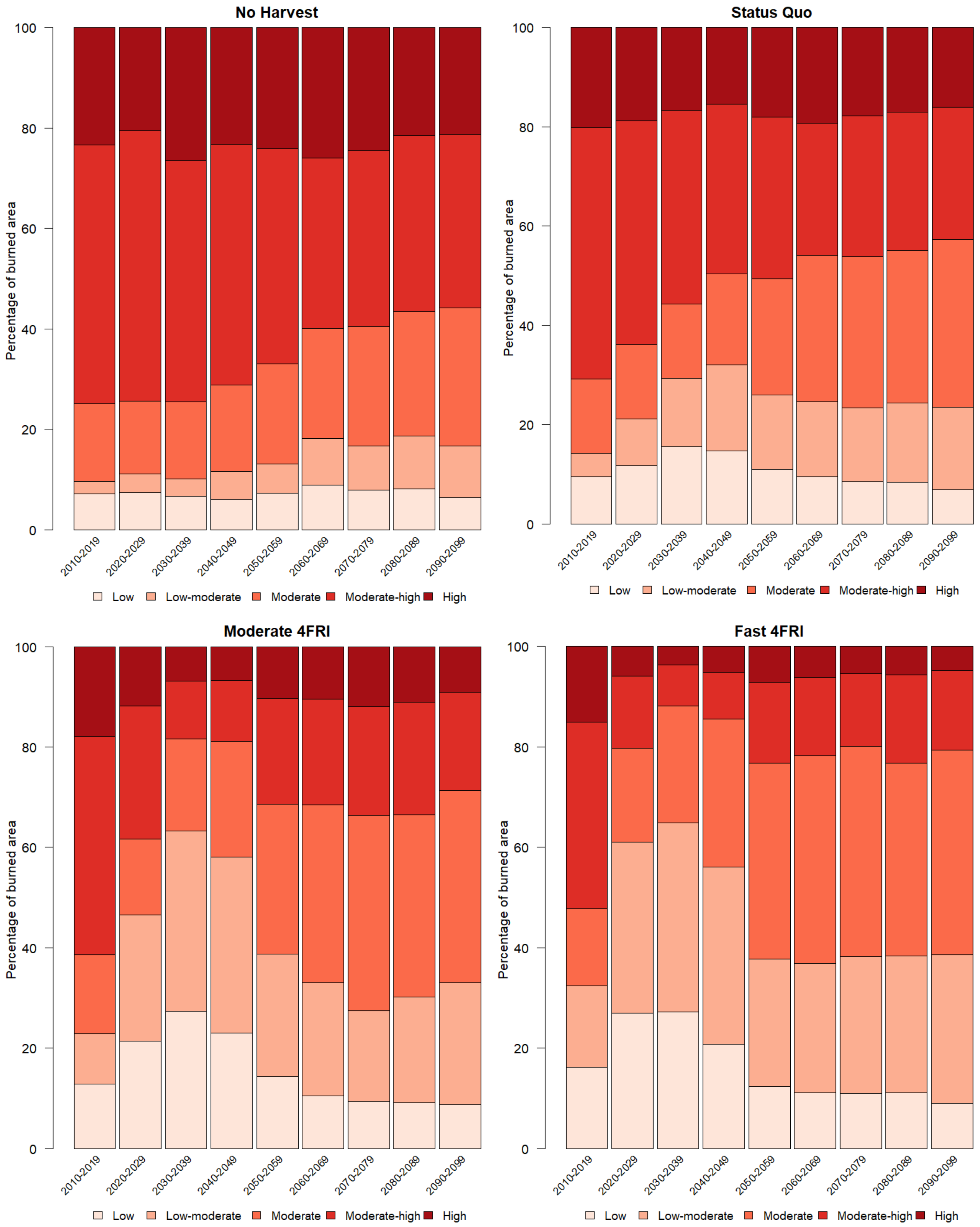

Figure S13. Percentage of burned area in each fire severity class in each scenario, averaged across all replicates and each decade, for the hot/drier climate model (IPSL CM5A LR)

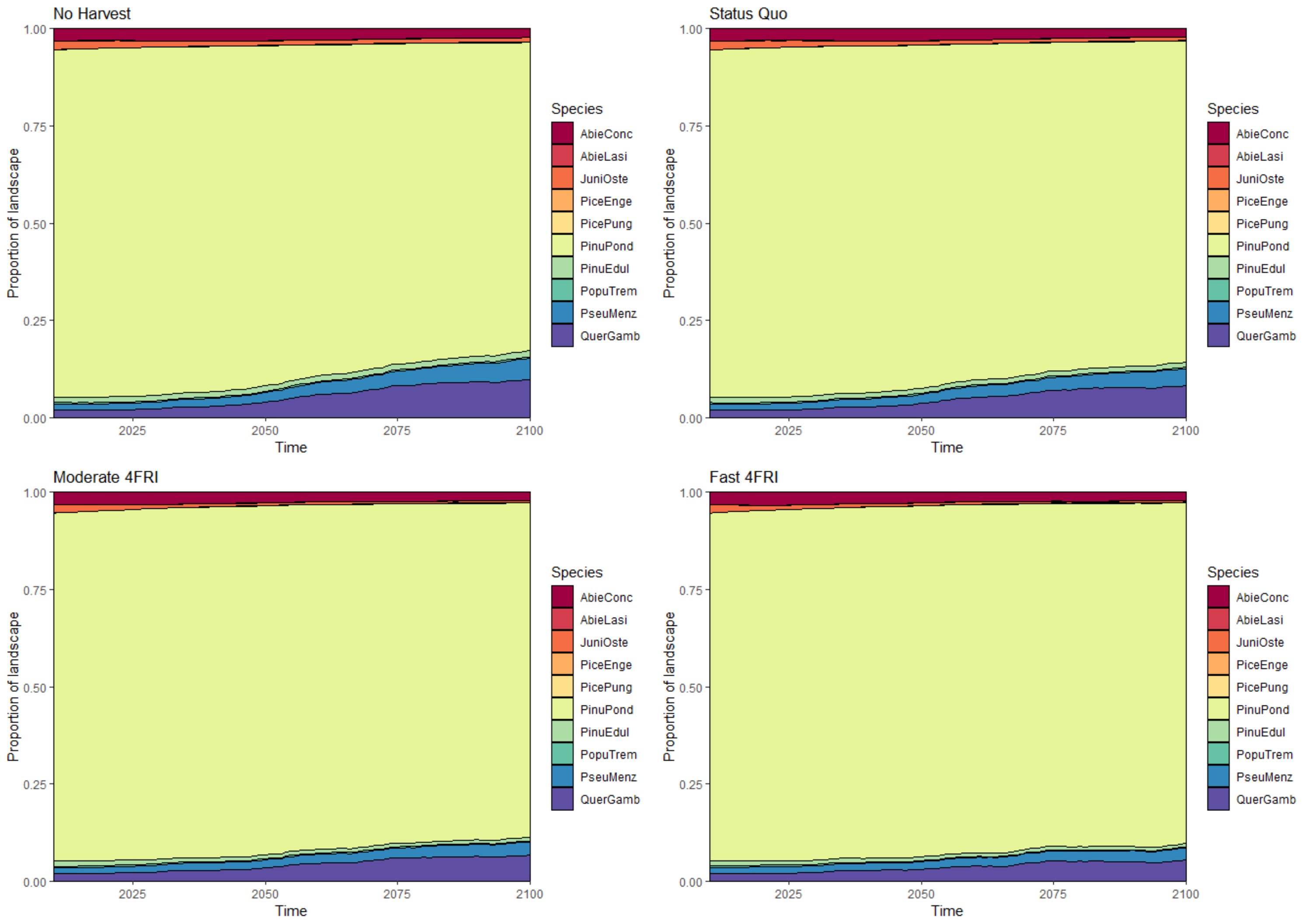

Figure S14. The proportion of landscape represented by each species in each scenario throughout the simulation period for the hot/drier climate model. AbieConc – *Abies concolor*, white fir; AbieLasi – *Abies lasiocarpa*, subalpine fir; JuniOste – *Juniperus*, juniper species; PiceEnge – *Picea engelmanii*, Englemann spruce; PicePung – *Picea pungen*, blue spruce; PinuPond – *Pinus ponderosa*, ponderosa pine; PinuEdul – *Pinus edulis*, pinyon pine; PopuTrem – *Populus tremuloides*, aspen; PseuMenz – *Pseudotsuga menziesii*, Douglas fir; QuerGamb – *Quercus gambelii*, Gambel oak
