# Supplementary material for: Large‐scale forest restoration stabilizes carbon under climate change in Southwest United States
Source: Ecol Appl. 2019 Aug 16;29(8):e01979. doi: 10.1002/eap.1979 (PMC6916600; doi:10.1002/eap.1979)
Supplement: Supplementary file 2 [file EAP-29-na-s002.pdf]

AppendixS2

Lisa A. McCauley, Marcos D. Robles, Travis Woolley, Robert M. Marshall, Alec Kretchun, and David F. Gori. **Large-scale forest restoration stabilizes carbon under climate change in Southwest United States.** *Ecological Applications*

Fate of Carbon Methods

Harvesting Equipment Emissions Calculations (Harvester, skidder, and processing equipment)

$$\textbf{MgC Emitted harvesting} = HARV\_RATE \times HARV\_AREA \times FUEL\_USE \times FUEL\_C \times 0.001$$

$HARV\_RATE$ = Local estimates of hours of machine time per hectare harvested in current restoration treatments (3.3 hrs/ha for harvester, 3.9 hrs/ha for skidding, 9.9 hrs/ha for processing)
$HARV\_AREA$ = Total Hectares Harvested
$FUEL\_USE$ = Liter/ha of fuel used. Based on equipment type, used most similar harvesting equipment (Markewitz, 2006) compared with local equipment used. We used 524 L/ha
FUEL_C = Constant of 0.7 kg C emitted per liter of fuel consumed by machinery (Markewitz, 2006)

Wood transportation Emissions Calculations

$$\textbf{MgC Emitted transportation to wood products facilities} = \frac{(HARV\_BIO \times PROD\_PORT)}{22.7^{\wedge}} \times \frac{DIST}{FUEL\_USE \times 2.77*} \times 0.001$$

$HARV\_BIO$ = Total Mg of biomass harvested
$PROD\_PORT$ = Estimated proportion of harvested material that is used for wood products (0.8) or residual biomass (0.2) products
$DIST$ = Total distance traveled to haul all biomass harvested (local data on biomass per truckload and distance from example restoration units to product facilities). We used roundtrip values of 355 miles for wood transport and 391 miles for biomass/chips transport

$FUEL\_USE$ = Total gallons of fuel used for transportation of biomass to wood products facilities = total miles of transportation / 6 MPG (local trucking data)

^ Mg of biomass per truck load
*Coefficient of kg C emitted per gallon of fuel used (U.S. EIA Carbon Emissions Coefficients https://www.eia.gov/environment/emissions/co2_vol_mass.php)

Wood Product Storage/Emissions Calculations

$$\boldsymbol{WOOD\_C} = HARV\_C \times WOOD\_PROP \times (1 - RESIDUAL\_WOOD\_PROP)$$

$$\textbf{Total MgC stored} = (WOOD\_C * 0.55)^{\frac{2100-year}{6}} + (WOOD\_C * 0.05)^{\frac{2100-year}{12}} + (WOOD\_C * 0.2)^{\frac{2100-year}{30}} + (WOOD\_C * 0.2)^{\frac{2100-year}{100}}$$

$$\textbf{MgC emitted from product decompostion} = WOOD\_C - Total\ MgC\ stored$$

$HARV\_C$ = Total Mg of biomass harvested * 0.5
$WOOD\_PROP$ = Estimated proportion of harvested material that goes to wood products (0.8)
$RESIDUAL\_WOOD\_PROP$ = Estimated proportion of wood product lost as emission during milling processes (0.5)

*Proportions of each product are : pallets – 55%; manufacturing – 5%; railroad ties – 15%; furniture – 5%; single family homes (post-1980) – 20%. Half-life estimates (the denominator in the exponent) from Skog (1998); Furniture and railroad ties have the same half-life so are combined in the equation

Total Carbon Storage and Emissions Wood Products Calculations

Total MgC stored (see above)

$$\textbf{Total MgC Emissions} = (HARV\_C \times RESIDUAL\_PROP) + (HARV\_C \times WOOD\_PROP\ x\ RESIDUAL\_WOOD\_PROP) + \textbf{MgC Emitted transportation} + \textbf{MgC Emitted harvesting} + \textbf{MgC emitted from product decomposition}$$

$HARV\_C$ = Total Mg of biomass harvested * 0.5
$RESIDUAL\_PROP =$ Estimated proportion of harvested material that is used for residual biomass products (0.2). Biomass products assumed to be emissions over study period.
$WOOD\_PROP =$ Estimated proportion of harvested material that goes to wood products (0.8)
$RESIDUAL\_WOOD\_PROP =$ Estimated proportion of wood product lost as emission during milling processes (0.5)

Markewitz, D. 2006. Fossil fuel carbon emissions from silviculture: Impacts on net carbon sequestration in forests. Forest Ecology and Management 236:153–161.

Skog, N. 1998. Carbon cycling through wood products: the role of wood and paper products in carbon sequestration. Forests Product Journal 48:9.
