# Supplementary material for: Large‐scale forest restoration stabilizes carbon under climate change in Southwest United States
Source: Ecol Appl. 2019 Aug 16;29(8):e01979. doi: 10.1002/eap.1979 (PMC6916600; doi:10.1002/eap.1979)
Supplement: Supplementary file 3 [file EAP-29-na-s003.pdf]

Lisa A. McCauley, Marcos D. Robles, Travis Woolley, Robert M. Marshall, Alec Kretchun, and David F. Gori. 2019. Large-scale forest restoration stabilizes carbon under climate change in Southwest United States. *Ecological Applications.*

Data S1

LANDIS-II input files.

Author(s) [of the material provided in DataS1.zip]

Lisa A. McCauley
The Nature Conservancy, Center for Science and Public Policy
Tucson, AZ U.S.A.
lisa.mccauley@tnc.org

Marcos D. Robles
The Nature Conservancy, Center for Science and Public Policy
Tucson, AZ U.S.A.
mrobles@tnc.org

Travis Woolley
The Nature Conservancy, Center for Science and Public Policy
Flagstaff, AZ U.S.A.
twoolley@tnc.org

Robert M. Marshall
The Nature Conservancy, Center for Science and Public Policy
Tucson, AZ U.S.A.
rmarshall@tnc.org

Alec Kretchun
Quantum Spatial
Portland, OR U.S.A.
aleckretchun@gmail.com

David F. Gori
School of Natural Resources & the Environment, University of Arizona
Tucson, AZ U.S.A.
dgori@email.arizona.edu

File list (files found within DataS1.zip)

DynamicFire.txt

DynamicFuels.txt

Harvest_fast.txt

Harvest_moderate.txt

Harvest_statusquo.txt

NECN-succession.txt

Species.txt

Description

DynamicFire.txt – data file used in LANDIS-II for the Dynamic Fire extension

DynamicFuels.txt - data file used in LANDIS-II for the Dynamic Fuel extension

Harvest_fast.txt - data file used in LANDIS-II for the Biomass Harvest extension

Harvest_moderate.txt - data file used in LANDIS-II for the Biomass Harvest extension

Harvest_statusquo.txt - data file used in LANDIS-II for the Biomass Harvest extension

NECN-succession.txt – data file used in LANDIS-II for the NECN Succession extension

Species.txt – data file used in LANDIS-II
